# Effectiveness of Lifestyle Interventions during Pregnancy on Preventing Gestational Diabetes Mellitus in High-Risk Women: A Systematic Review and Meta-Analyses of Published RCTs

**DOI:** 10.3390/jcm12227038

**Published:** 2023-11-10

**Authors:** Georgios I. Tsironikos, Petros Potamianos, George E. Zakynthinos, Vasiliki Tsolaki, Athina Tatsioni, Alexandra Bargiota

**Affiliations:** 1 Department of Medicine, University of Ioannina, University Campus, 45110 Ioannina, Greece; g.tsironikos@uoi.gr; 2 Department of Gastroenterology, University Hospital of Larissa, Faculty of Medicine, University of Thessaly, Mezourlo, 41335 Larissa, Greece; petpot92@gmail.com; 3 3rd Department of Cardiology, “Sotiria” Chest Diseases Hospital, Medical School, National and Kapodistrian University of Athens, 11527 Athens, Greece; gzakynthinos2@gmail.com; 4 Department of Critical Care, University Hospital of Larissa, Faculty of Medicine, University of Thessaly, Mezourlo, 41335 Larissa, Greece; 5 Department of Research Unit for General Medicine and Primary Health Care, Faculty of Medicine, University of Ioannina, University Campus, 45110 Ioannina, Greece; atatsion@uoi.gr; 6 Department of Internal Medicine-Endocrinology, University Hospital of Larissa, Faculty of Medicine, University of Thessaly, Mezourlo, 41335 Larissa, Greece; abargio@med.uth.gr

**Keywords:** diet, nutrition, exercise, physical activity, gestational diabetes mellitus

## Abstract

Background: Until now, it is uncertain whether lifestyle interventions during pregnancy can prevent gestational diabetes mellites (GDM) in high-risk pregnant women. Objective: This study aims at investigating the effectiveness of dietary interventions and/or exercise interventions during pregnancy for preventing GDM in high-risk pregnant women. Materials and Methods: Eligible randomized controlled trials (RCTs) were selected after a search in CENTRAL, Scopus, and PubMed. Synthesis was performed for the outcome of GDM in women with any identified GDM risk factor. Separate meta-analyses (MA) were performed to assess the efficacy of either nutrition or physical activity (PA) interventions or both combined compared with standard prenatal care for preventing GDM. Subgroup and sensitivity analyses, as well as meta-regressions against OR, were performed to assess potentional heterogeneity. Overall quality, the quality of RCTs, and publication bias were also evaluated. Results: A total of 13,524 participants comprising high-risk pregnant women in 41 eligible RCTs were analyzed for GDM. Women receiving only a nutrition intervention during pregnancy were less likely to experience GDM compared with women following standard prenatal care. Among 3109 high-risk pregnant women undergoing only dietary intervention for preventing GDM, 553 (17.8%) developed GDM; however, the result of the MA was marginally not significant (OR 0.73, 95%CI 0.51, 1.03; *p*-value 0.07), (Q 21.29, *p*-value 0.01; I^2^ 58% (95%CI 10, 78%)). Subgroup analyses demonstrated an effect for studies that were conducted in Great Britain (OR 0.65, 95%CI 0.49, 0.81; *p*-value 0.003), and in Spain (OR 0.50, 95%CI 0.27, 0.94; *p*-value 0.03), for studies with forms of the Mediterranean diet as the intervention’s component (OR 0.61; 95%CI 0.46, 0.81; *p*-value 0.0005), and for studies including a motivation arm in the intervention (OR 0.71, 95%CI 0.58, 0.87; *p*-value 0.0008). Among 2742 high-risk pregnant women being analyzed for GDM outcome after receiving only an exercise intervention, 461 (16.8%) were diagnosed with GDM. Women after receiving PA intervention were less likely to develop GDM (OR 0.64, 95%CI 0.51, 0.80; *p*-value < 0.0001), (Q 11.27, *p*-value 0.51; I^2^ 0% (95%CI 0, 99%)). Finally, 1308 (17%) cases of GDM were diagnosed among 7673 high-risk pregnant women undergoing both diet and PA intervention. Women in the group of mixed lifestyle intervention had a significant reduction in incidence of GDM (OR 0.70, 95%CI 0.55, 0.90; *p*-value 0.005), (Q 50.32, *p*-value < 0.0001, I^2^ 66%, (95% CI 44, 79%)). Conclusions: The results of this study support the efficacy of lifestyle interventions during pregnancy for preventing GDM in high-risk women if an exercise component is included in the intervention arm, either alone, or combined with diet. A combined lifestyle intervention including physical exercise and a Mediterranean diet accompanied by motivation support may be considered the most effective way to prevent GDM among high-risk women during pregnancy. Future research is needed to strengthen these findings.

## 1. Introduction

According to the latest guidelines of the American Diabetes Association (ADA), gestational diabetes mellites (GDM) is a type of diabetes mellitus (DM) that is recognized after the first trimester of pregnancy [1,2]. It is the most common gestational and childbirth complication [3,4]. Independently of the applied diagnostic criteria, the incidence of GDM is increasing in worldwide [5,6]. Therefore, it is an important disease that affects pregnancies [7], increasing the incidence of both short-term and long-term unfavorable health circumstances [8].

Many risk factors have been implicated in GDM including non-white race or ethnicity [9], Hispanic, Middle Eastern, Southern Asian, Polynesian, and African ethnic groups [3,10,11], low-middle income (LMI) and low education level [12], advanced maternal age (≥35 years) [8,13], maternal smoking [14], westernized diet [10], diets with low fiber concentrations and with a high glycaemic load (GL) [9,15], diets with increased consumption of saturated fats and decreased consumption of polyunsaturated fats [5], physical inactivity [9,15], increasing and high parity [9,15], pre-existent overweight or obesity [11,16], excessive gestational weight gain (GWG) [5,10,17], maternal adiposity [17], family history of first-degree relatives with DM [8,10,15], maternal high or low birth weight [9], history of GDM [10,15], history of macrosomia (birthweight ≥ 4000 g) [8,15], history of congenital abnormalities [18], history of abortion [18] or recurrent abortions [14], history of preterm delivery [18], previous fetal death [19], previous stillbirth [14], polycystic ovarian syndrome (PCOS) [10,15], history of hypertension (HY) or pregnancy-associated high blood pressure (BP) [14], abnormal lipid metabolism [8], and persistent glucosuria [14]. Previous obesity, HY or hypertriglyceridaemia increase metabolic risk in pregnancy [20]. 

Several interventions to mitigate hyperglycemia have been suggested, the main ones being pharmacological, PA and lifestyle [21]. However, the research question of whether GDM could be prevented by interventions during pregnancy or before pregnancy remains unanswered [22,23]. Physical activity (PA) may be protective against T2DM, although the data regarding PA and GDM are less extensive and less convincing [24]. Yet, there is insufficient evidence that lifestyle interventions are effective in preventing GDM [25]. 

According to a recent systematic review (SR) and meta-analysis (MA), any intervention based on diet or PA or both of them, resulted in significantly less occurrence of GDM [26]. Additionally, a recent network MA demonstrated that GDM could be prevented with the implementation of exercise plus probiotic interventions, whereas dietary only, or dietary plus PA interventions did not alter the GMS incidence [1]. However, a previous SR and MA did not find any benefit with diet, or exercise, or a mixed approach for reducing GDM risk [27]. Similarly, three SRs and MAs assessing lifestyle interventions during pregnancy including diet and PA reported no effect on decreasing the outcome of GDM in obese or overweight women [23,28,29]. 

Preventing GDM is a priority in pregnancy [21]. Moreover, pregnancy may represent a good time period for lifestyle changes as pregnant women have strong willingness to improve the health benefits for them and for their offsprings [28]. In this SR and MA, we aim to summarize the most recent evidence regarding the efficacy for preventing GDM among high-risk women of any lifestyle interventions, including either nutrition or PA interventions, or combined diet-plus-exercise interventions that are implemented during the gestational period. 

## 2. Materials and Methods

Our study was pre-registered in the Open Science Framework (OSF) (Registration DOI 10.17605/OSF.IO/UMG28, https://osf.io/uvr9d/registrations (accessed on Monday 29 August 2022) 1). This SR was performed according to the PRISMA extension for complex intervention guidelines [30].

### 2.1. Search Strategy

PubMed, Cochrane Library Central Register of Controlled Trials (CENTRAL), and Scopus were searched (from inception of data to August 2022). The search strategy for Pubmed included keywords related to diet, nutrition, exercise, PA, and GDM combined with the Cochrane Collaboration search algorithm for randomized controlled trials (RCTs). CENTRAL and Scopus were searched systematically using the same keywords (Table S1). Based on title and/or abstract, the full text was retrieved for unclear items or potential eligibility. One investigator (GIT) screened all the databases. A second investigator (AB) checked the items for which the first investigator (GIT) could not reach a decision. Discrepancies were resolved through consensus. 

### 2.2. Eligibility Criteria

The PICO (population, intervention, comparator, and outcome) approach was used for selecting eligible trials. RCTs in English language, including high-risk pregnant women for GDM with any identified risk factor were accepted. RCTs evaluating the risk factor of overweight and/or obesity among participants via BMI undivided and as a total group without participants’ stratification were considered as eligible. RCTs appraising any type of active lifestyle intervention of diet alone, exercise alone, or both during pregnancy compared with standard antenatal care were included. Trials involving the outcome of GDM being diagnosed by any recommended modality were chosen. 

Protocols of RCTs, pilot RCTs, secondary analyses, and abstracts from conference proceedings were excluded. 

### 2.3. Data Extraction

Two independent researchers (GIT and PP) extracted the data. Discrepancies were resolved by consensus. When necessary, a third arbitrator (AB) contributed to the final decision. The extracted items were the first author’s name, publication year, country where performed, type of RCT, number of centers for multicentered trials, study duration, drop-out rate, sample size, women’s mean age, women with low education level according to authors’ assessment of any eligible trial, GDM risk factors, the type of intervention and the care for provided in the control group, as well as any potential reported side-effects for both the experimental, and comparator groups. The diagnostic methods and time period of screening for GDM were also recorded. The Consensus on Exercise Reporting Template (CERT) tool for complex interventions was used to evaluate exercise programs in RCTs applying only PA in the intervention arm [31]. Finally, the number of patients with GDM being diagnosed by any method was captured as an outcome, separately in any group. 

### 2.4. Quality Assessment of the Studies and Rating of Overall Evidence

To evaluate the quality of the eligible RCTs, the risk of bias tool proposed by the Cochrane Collaboration was used [32]. In addition, overall evidence was rated through the Grading of Recommendations, Assessment, Development and Evaluation (GRADE) framework (GRADEpro, Version 3.6.1. McMaster University, 2011) [33].

### 2.5. Statistical Analysis

The main analyses included all available data. The significance level for Cochran’s Q statistic was set at *p*-value < 0.1, and for the rest of the analyses, at *p*-value < 0.05 [34]. SPSS 22.0 (SPSS, Inc., Chicago, IL, USA), Stata Statistical Software 10.1 (Stata, College Station, TX, USA), and Review Manager 5.4.1 (Cochrane Collaboration, London, UK) were used for the analyses.

Both fixed effects (FE) and random effects (RE) MA were performed to combine the GDM events. Heterogeneity between studies was assessed by Cochran’s Q statistic [34]. Heterogeneity was measured with the I^2^ index (<25%, low; 25–49%, moderate; 50–74%, large; >75%, very large) [35]. In case of large heterogeneity, the results were synthesized by RE (odds ratio (OR) with 95% CI) [34].

Separate analyses [36] were performed for studies based on the studies’ performance country, for studies with more than 10% of participants with a low education level, and for studies evaluating or not overweight or obesity as a GDM risk factor. Moreover, separate analyses were conducted for studies with an intervention duration of more than 20 weeks and less than 20 weeks, and for trials including a Mediterranean diet as a component of dietary interventions or including any dietary intervention. Finally, subgroup analyses included trials assessing both a motivation component in the intervention, and trials not including motivation. The effect of the RCTs with the largest sample size was also estimated by their exclusion in sensitivity analyses [36]. Additionally, sensitivity analyses were also performed for studies with low attrition bias [36]. Μeta-regression analyses on GDM OR were conducted with the effect of baseline risk, and study duration as covariates [36,37]. Publication bias was assessed via the visual analysis of a funnel plot [38]. The statistical test of Egger was also performed for publication bias assessment [39].

## 3. Results

### 3.1. Eligible Studies

**Figure 1:**
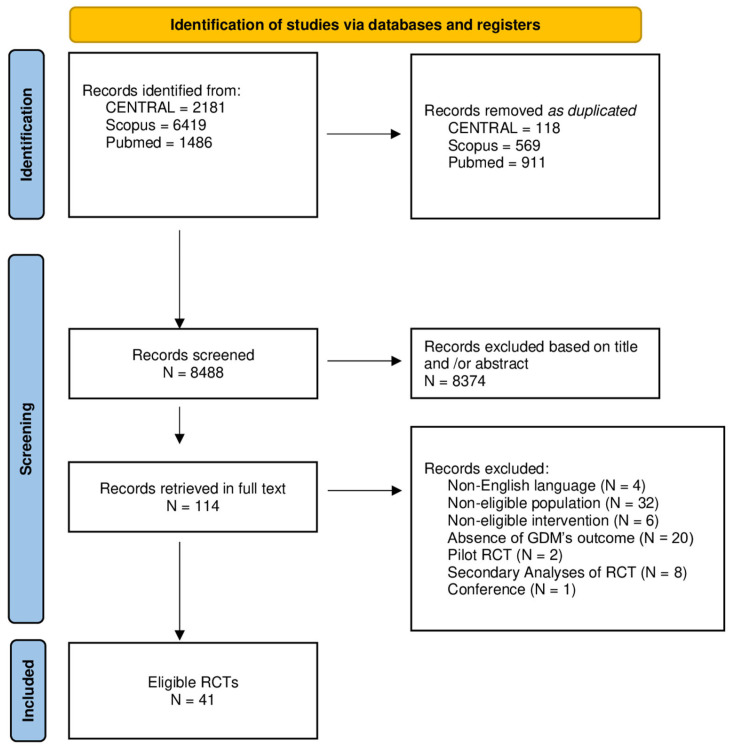
Flow chart of procedures for selecting studies.

The search yielded 10,086 items (2181 in CENTRAL, 6419 in Scopus, and 1486 in PubMed). A total of 1598 items were excluded as duplicated. Out of the 8488 remaining items, 8374 were excluded as non-relevant based on the title, or abstract. Thus, 114 papers were retrieved in full text. Out of the 114 articles, 73 were excluded: 4 studies were published in a non-English language; 32 studies did not include an eligible population; 6 studies included a non-eligible intervention; 20 trials did not report the onset of GDM as an outcome; 2 papers reported a pilot RCT; 8 papers included secondary analyses of RCTs; and 1 paper was retrieved from a conference. Finally, this study included 41 eligible published RCTs. Specifically, 10 of them reported diet-only interventions, 13 exercise-only interventions, and 18 diet-plus-exercise interventions (Figure 1).

### 3.2. Characteristics of Eligible Studies

Ten eligible RCTs were identified with an intervention of diet only. These were published between 2011 and 2022 (Table 1). Four of them were conducted in Europe (one each in Spain, the United Kingdom (UK), Finland and Ireland) [3,20,40,41]. Another three were conducted in Oceania (two in Australia and one in New Zealand) [42,43,44], two in China [16,45], and one study was conducted in the United States of America (USA) [4] (Table 1). Two trials were multi-centered (two and five recruited centers, respectively) [20,45] (Table 1). The study designs were parallel [3,4,16,20,40,41,42,43,45], except for one crossover RCT [44] (Table 1). The study durations ranged from 10 to 48 months, although the duration was not reported in two studies [40,43] (Table 1). All the RCTs had a drop-out ratio below 30% (Table 1).

Thirteen RCTs with exercise intervention alone, were considered as eligible. They were published between 2011 and 2022 (Table 2). Six trials were conducted in Europe (three in Spain, one in Ireland, one in Norway, and another one in the Netherlands) [46,47,48,49,50,51], four in the Americas (two in the USA, one in Brazil, one in Canada) [52,53,54,55], two in Oceania (one in Australia, and the other in New Zealand) [56,57], and one in China [58] (Table 2). All the trials had parallel designs, and two of them were multicentered [49,51] (three and five centers, respectively) (Table 2). The duration of the studies varied between 19 and 60 months (Table 2). The drop-out ratio was less than 20% in eleven RCTs [46,47,49,50,51,52,53,55,56,57,58], and more than 30% in the remaining two, with drop-out ratios estimated at 31.9% [54], and 41.1% [48], respectively (Table 2).

Eighteen eligible trials implemented a complex intervention of diet and exercise. Their publication years were from 2011 to 2022 (Table 3). Seven were performed in Europe (two in Finland, two in Italy, one in Ireland, one in Denmark, and one in the UK) [5,6,17,59,60,61,62], seven in Asia (six in China, and one in the United Arab Emirates (UAE)) [7,8,10,13,18,63,64], two in Australia [11,65], and two in North America (one in the USA, and one in Canada) [66,67] (Table 3). Seven of these trials recruited participants using a multi-centered approach with the number of centers between 2 and 14 [5,6,11,59,60,65,66], and 11 of them were single-centered [7,8,10,13,17,18,61,62,63,64,67] (Table 3). Their designs were parallel [6,7,8,10,11,13,17,18,59,60,61,62,63,64,65,66,67], apart from one cluster RCT [5] (Table 3). The duration of the trials ranged between 5 and 71 months (Table 3). Sixteen trials had a drop-out ratio less than 30% [5,6,7,8,10,11,13,17,18,59,60,61,63,65,66,67], one trial had a rate of 31.4% [62], and one study did not provide data [64] (Table 3). 

## 4. Characteristics of Participants

A total of 3690 pregnant women with high risk of GDM participated in the eligible trials with only a dietary intervention arm (1841 in intervention groups, and 1849 in control groups). Their mean age varied from 31.7 to 21.7 years for women in the intervention groups (*n* = 1744), and from 36.9 to 22.0 years for women in the control groups (*n* = 1754) (Table 4). One study reported the mean age of participated women at 28.3 years in the intervention group (*n* = 63) and 29.5 in control group (*n* = 61) without a standard deviation (SD) [43], and another study did not report data regarding age [40] (Table 4). Three trials provided data about education level [3,42,44] (Table 4). The percentage of women with a low education level ranged between 10.5% and 30.2% in the experimental groups, and between 7.8% and 29.8% in the comparator groups (Table 4). Overweight or obesity was a GDM risk factor identified in eight RCTs [4,16,20,40,42,43,44,45] (Table 4). Family history of DM or history of GDM were included in two RCTs [4,40] (Table 4). Two RCTs also investigated the history of previous macrosomia [40,41] (Table 4). Advanced maternal age was mentioned in one RCT [40] (Table 4). Hispanic origin was examined in one trial [3] (Table 4). Chronic HY or abnormal lipid metabolism were appraised in another study [20] (Table 4).

In total, 3073 pregnant women with high risk for GDM participated in studies implementing only exercise (1532 in intervention groups, and 1541 in control groups). Their mean age ranged from 22.9 to 38 years in the intervention groups (*n* = 1351), and from 20.3 to 37.7 years in the control groups (*n* = 1357) (Table 5). One study reported only the range of age of participating women (18–40 years; *n* = 290) [55] (Table 5). The percentage of women with a low education level varied between 2.3% and 40% in intervention groups, and between 7% and 36% in the control groups (Table 5). Four trials did not mention the women’s education level [47,54,56,57], and one trial reported percentages of women who did not receive university education [49] (Table 5). Overweight or obesity were reported as risk factors for GDM in eight RCTs [47,50,51,52,53,55,57,62]. A sedentary lifestyle was reported as a risk factor in four RCTs [46,48,49,54], family history of diabetes and previous GDM in two RCTs [51,55], and history of macrosomia in one RCT [51] (Table 5).

A total of 8532 pregnant women high risk for GDM participated in RCTs applying both diet and exercise interventions (4317 in intervention groups, and 4215 in control groups). Their mean age varied between 23.9 and 41 years in the experimental groups (*n* = 4137), and between 20 and 40.5 years in the comparator groups (*n* = 4035) (Table 6). One trial reported a mean age of 29 years with ranges for both the intervention (*n* = 180) and control arms (*n* = 180) [59] (Table 6). The percentage of women with a low education level ranged from 2.7% to 40.6%, and from 2.2% to 47.7%, in intervention and control group, respectively (Table 6). Eight studies did not report data regarding the women’s education level [7,8,18,60,63,64,65,67] (Table 6). The most frequently appearing GDM risk factor was overweight or obesity, which was reported in 17 out of 18 eligible trials [5,6,7,8,10,11,13,17,18,59,60,61,62,63,65,66,67] (Table 6). Next, history of GDM was included in six trials [5,6,8,10,13,18], and family history of diabetes was evaluated in five trials [5,8,10,13,18] (Table 6). Furthermore, advanced maternal age was examined in five trials [5,8,13,18,64] (Table 6). In addition, history of macrosomia was investigated in four trials [5,8,10,13], and history of PCOS was assessed in three studies [10,18,63] (Table 6). Finally, high-risk ethnicities were identified in two trials [10,11], and history of abnormal lipid metabolism, and elevated fasting plasma glucose (FPG) in early pregnancy were investigated in one study [8] (Table 6).

### 4.1. Characteristics of Interventions

Dietitians were the providers of dietary interventions in five eligible RCTs [3,16,40,41,45]; in particular, one trial reported a clinical nutritionist as the provider [41] (Table 7). In addition, in three RCTs, dietitians were assisted by nurse practitioners [4], trained researchers [20], and health workers [44] (Table 7). Midwifes [42], and a food technologist [43] provided the nutrition intervention in the remaining two RCTs [42,43] (Table 7). One trial reported an exact time period of intervention at 10 weeks [20] (Table 7). The mean intervention durations in ascending order in the remaining trials (in weeks) were as follows: 17.7 (15.4–20) [16], 19 (16–22) [41], 25.5 (22.4–28) [45], 28 [40], and 30 (28–32) [3] (Table 7). Three RCTs reported mean intervention durations (in weeks) of >14 [4], >16 [42], and ≥19 [45] (Table 7). One trial provided no data on the duration of the intervention [43] (Table 7). The nutritional interventions varied in terms of delivery, components, motivation, assessment and side effects/adverse events, and are described in detail in Table 7. 

Exercise was the exclusive intervention in 13 eligible trials. Researchers were the providers of the exercise in four studies [47,49,54,62], physiotherapists in three studies [50,51,52], and exercise physiologists in two trials [56,57] (Table 8). In the remaining studies, the intervention was provided by fitness instructors [48], kinesiologists [53], health educators [55], and fitness specialists with the assistance of an obstetrician [46] (Table 8). The duration of exercise varied across studies. According to the RCTs reporting an exact period of intervention duration in weeks, the durations in increasing order were 10 [55], 12 [53], 14 [56], 15 [57], and 25 [51] (Table 8). Other trials reported the mean intervals of exercise intervention in weeks. The intervals in increasing order were 17 (12–22) [52], 23 (22–28) [54], 24.5 (21–28) [50], 26.4 (25 + 1/7–27 + 4/7) [47], 27 (25–29) [62], 27.5 (26–29) [46], 28.5 (27–30) [48], and 29.5 (29–30) [49] (Table 8). One trial reported an additional PA until the sixth week postpartum [47] (Table 8). The core of exercise programs in each eligible trial included aerobic PA (Table 8). The exercise programs, providers, delivery, components, motivation, assessment, and potential side effects are reported in Table 8.

Eighteen eligible RCTs involved exercise in addition to dietary interventions. Dietitians provided the combined intervention in six of them [10,58,61,63,66,67], researchers in three of them [17,18,64], health trainers in another two [11,60], and nurses in one trial [5] (Table 9). In six studies, the dietitians provided the intervention in collaboration with physiotherapists [59], trained research assistants [65], study nurses [6], exercise instructors [13], clinical nutritionists [7], or exercise experts and nurses [8] (Table 9). The durations of the interventions from shortest to longest reported exactly in weeks were 8 [60], 12 [10], and 20 [61] (Table 9). The intervention duration in trials which reported mean week ranges were in ascending order as follows: 12 (10–14) [8], 13 (12–14) [11], 13 (10–16) [67], 13.1 (12.2–14) [66], 19.5 (17–22) [17], 21.7 (19.8–23.6) [6], 23 (20–26) [59], 23 (20–26) [65], 25.5 (24–27) [58], 27 (25–29) [5], and 30 (28–32) [63]. One trial reported an intervention period > 12 weeks [13], another study > 14 [7], and another one > 18 weeks [18]. One trial did not report data on study duration [64]. All the interventions included a motivation arm (Table 9). Details on the dietary and exercise arms of the interventions, as well as potential side effects are reported in Table 9.

### 4.2. Description of Exercise Intervention in Eligible Trials

Exercise is reported based on the CERT tool for eligible trials implementing only exercise intervention [31] (Table S2). All trials reported measures of adherence, dosage of PA, measures of how well the intervention was delivered, and documentation or management of possible adverse events [46,47,48,49,50,51,52,53,54,55,56,57,62] (Table S2). Four trials included a home exercise component [50,52,56,57] (Table S2). Two trials provided description of the exercise capable of replication and reproduction [48,57] (Table S2).

One trial did not report any equipment type used during exercise [52] (Table S2). One other trial did not mention the provider [49] (Table S2). Two studies did not describe whether PA was performed individually or in a group [55,62], and one of them did not mention the existence of supervision or not [55] (Table S2). No data regarding the motivation component of intervention were found in five RCTs [48,49,54,56,62] (Table S2). One trial did not introduce decision rules for progressing exercise program [52]. The location of the exercise was not clarified in one trial [54] (Table S2). Seven out of thirteen studies did not determine decision rules for the starting level of exercise [46,48,49,50,52,56,57] (Table S2).

The exercise interventions were tailored to individuals in seven trials [49,50,51,53,56,57,62], and were generic in the remaining six RCTs [46,47,48,52,54,55] (Table S2). 

### 4.3. GDM Diagnosis

Diagnosis of was based on the glucose values of a two-hour 75 g oral glucose challenge test (OGTT) in seven out of ten eligible trials with dietary interventions [3,16,20,40,43,44,45] (Table S3). The diagnosis of GDM was based on the glucose values of a two-step OGTT in the remaining three trials [4,41,42], including a one-hour 50 g OGTT plus two-hour 75 g OGTT in one trial [42], and a one-hour 50 g OGTT plus three-hour 100 g OGTT in the other two trials [4,41] (Table S3). A majority (nine trials) reported that the diagnostic tests were performed after the 24th gestational week [3,4,16,20,41,42,43,44,45]; however, one study mentioned that the participating women underwent a two-hour 75 g OGTT in early pregnancy (between 8 and 12 weeks of pregnancy) [40] (Table S3). 

Six among thirteen eligible trials including exercise intervention reported glucose values of two-hour 75 g OGTT to diagnose GDM [46,47,50,56,57,58] (Table S4). A one-step approach to diagnosing GDM was also applied in another RCT with a three-hour 100 g OGTT [54] (Table S4). Two trials reported a combined one-hour 50 g OGTT plus three-hour 100 g OGTT to screen for GDM [48,55] (Table S4). Values of FPG and hemoglobin A1c (HbA1c) were adopted by one other study [51] (Table S4). Nine trials reported that screening was performed after the 24th week of gestation [46,47,48,50,51,55,56,57,58] (Table S4). Moreover, one trial reported that screening for GDM was also performed during 32th week of gestation, in addition to the 24th [51] (Table S4). Three studies did not report data concerning either the diagnostic modality for GDM, or the gestational week at which the tests were performed [49,52,53] (Table S4). Additionally, the time interval of testing was not reported in another study [54] (Table S4). 

The diagnosis of GDM was determined by the glucose values of a two-hour 75 g OGTT in 16 eligible trials involving diet-plus-exercise interventions [5,6,7,8,10,11,13,17,18,59,60,61,62,63,65,67] (Table S5). Screening for GDM was conducted by a two-step approach of one-hour 50 g OGTT plus three-hour 100 g OGTT in two RCTs [64,66] (Table S5). OGTTs were performed after the 24th week in all studies [5,6,7,8,10,11,13,17,18,59,60,61,62,63,64,65,66,67]. In addition, six trials reported extra time intervals of gestational weeks of GDM screening [11,59,61,62,65,66] (Table S5). In particular, in one trial, two extra periods of GDM screening during early and late pregnancy were described (at 12–14, and 34–36 weeks of gestation, respectively) [59] (Table S5). In the remaining five trials, the time intervals of additional GDM screening included early pregnancy, specifically at 12–15 [11], 16–18 [61], 12–14 [65], 16–18 [62], and 8–15 [66] weeks of pregnancy (Table S5).

### 4.4. Effectiveness of Lifestyle Interventions during Pregnancy

**Figure 2:**
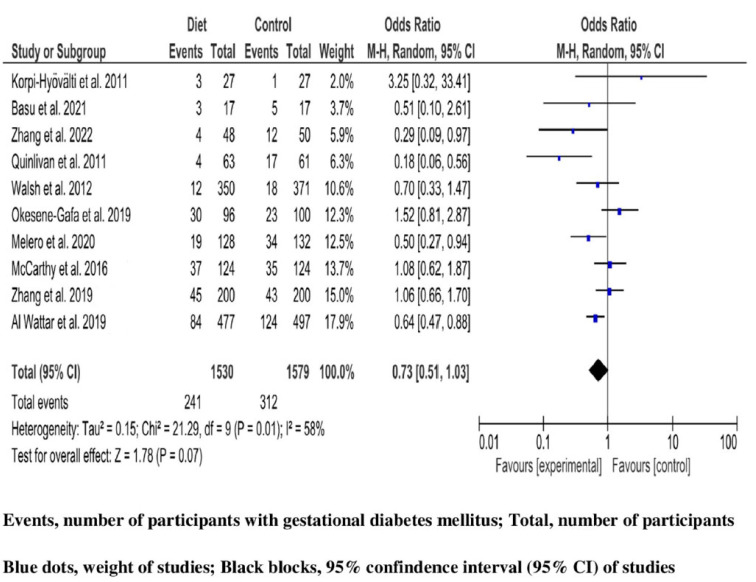
Dietary intervention and the risk of gestational diabetes mellitus in high-risk pregnant women. Studies presented by weight contributing to the meta-analysis-blue dots, in odds ratio (OR) estimates corresponding to their 95% confidence interval (95% CI)-black blocks. Summary of OR by random effect model demonstration, as well as metrics of heterogeneity [3,4,16,20,40,41,42,43,44,45].

After drop-out, a total of 3109 (1530 in intervention, and 1579 in control groups) pregnant women with high risk for GDM receiving dietary intervention were analyzed for GDM outcome. Among them, 553 (17.8%) developed GDM (241 (15.7%) in the intervention group, and 312 (19.7%) in the control group). Combining studies, heterogeneity across them was significant (Q 21.29, *p*-value 0.01), and variability was large (I^2^ 58% (95%CI 10, 78%)). Therefore, an RE model was selected for synthesizing the effect of dietary interventions. Women receiving nutrition intervention during pregnancy were less likely to develop GDM compared with women following standard prenatal care; however, the result of the MA was marginally not significant (OR 0.73, 95%CI 0.51, 1.03; *p*-value 0.07) (Figure 2).

In contrast to the summary OR, a significant effect was shown when analyses were limited to (a) studies that were performed in Great Britain (OR 0.65, 95%CI 0.49, 0.81; *p*-value 0.003), and in Spain (OR 0.50, 95%CI 0.27, 0.94; *p*-value 0.03); (b) to studies including overweight or obesity as a GDM risk factor (OR 0.78, 95%CI 0.64, 0.96; *p*-value 0.02), or studies where BMI was not included among GDM risk factors (OR 0.57, 95%CI 0.36, 0.93; *p*-value 0.02); (c) to studies including forms of the Mediterranean diet as an intervention component (OR 0.61 95%CI 0.46, 0.81; *p*-value 0.0005); and finally, (d) to studies with a motivation component in the intervention (OR 0.71, 95%CI 0.58, 0.87; *p*-value 0.0008). The test of difference was not statistically significant for any subgroup analysis (Table S6). In the sensitivity analysis evaluating the effect of the RCT with the largest sample size [20], the OR remained non-significant (OR 0.73; 95%CI 0.48, 1.13; *p*-value 0.16) (Table S6). Due to the small number of trials and potentional variability among studies in subgroup analyses, significant results should be interpreted cautiously. Meta-regression analyses with baseline risk, and study duration as covariates did not show a statistically significant effect on the summary OR (Table S7).

**Figure 3:**
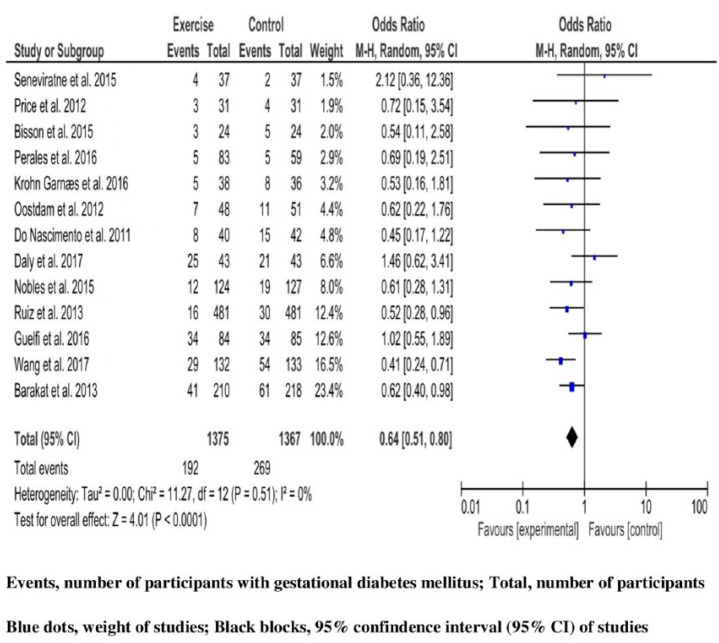
Exercise intervention and the risk of gestational diabetes mellitus in high-risk pregnant women. Meta-analysis of eligible studies with random effect model. Studies are presented according to their weight contributing to the synthesis-blue dots. Estimates of odds ratio (OR) with 95% confidence interval (95% CI) of each trial-black blocks, and overall OR with measures of heterogeneity [46,47,48,49,50,51,52,53,54,55,56,57,58].

461 reports represent a 16.8% level of occurrence of diagnosed GDM (including 192 (14%) in the intervention group, and 269 in the control group (19.7%)) among 2742 high-risk pregnant women (1375 in the intervention group, and 1367 in control group) analyzed for gestational diabetes outcome, after drop-out. Heterogeneity across studies was non-significant (Q 11.27, *p*-value 0.51); however, variability could not be excluded as the upper limit for I^2^ is greater than 75% (I^2^ 0% (95%CI 0, 99%)). Therefore, the results of the synthesis are presented through the FΕ model. The result of the MA revealed a significant reduction in GDM outcomes for women participating in the exercise intervention group compared with those participating in the standard care group (OR 0.64, 95%CI 0.51, 0.80; *p*-value < 0.0001) (Figure 3).

Based on subgroup analyses, the summary OR remained significant when analyzing separately (a) studies that were performed in Spain (OR 0.59, 95%CI 0.42, 0.84; *p*-value 0.003), and in China (OR 0.41, 95%CI 0.24, 0.71; *p*-value 0.001); (b) studies including participant women with low education level at a percentage more than 10% (OR 0.54, 95%CI 0.42, 0.69; *p*-value < 0.00001); (c) studies considering overweight or obesity as a GDM risk factor (OR 0.60, 95%CI 0.44, 0.82; *p*-value 0.001), or without including BMI as a risk factor (OR 0.68, 95%CI 0.50, 0.91; *p*-value 0.01); (d) studies applying an intervention lasting more than 20 weeks (OR 0.60, 95%CI 0.48, 0.75; *p*-value < 0.0001); and (e) studies applying a motivational component in the exercise intervention (OR 0.69, 95%CI 0.51, 0.92; *p*-value 0.01), or without a motivation arm (OR 0.59, 95%CI 0.43, 0.81; *p*-value 0.001). However, the test of difference was non-significant for any subgroup analysis (Table S8). Investigating the effect of the RCT with the largest sample size [49], and the RCT with a high attrition bias [48] (drop-out ratio 41.1%) in sensitivity analyses, the summary OR remained significant; (OR 0.66, 95%CI 0.52, 0.80; *p*-value 0.0005), and (OR 0.64, 95%CI 0.51, 0.80; *p*-value 0.0001), respectively (Table S8). Baseline risk, and study duration as covariates in meta-regression analyses did not have a significant effect on the summary OR (Table S9). 

**Figure 4:**
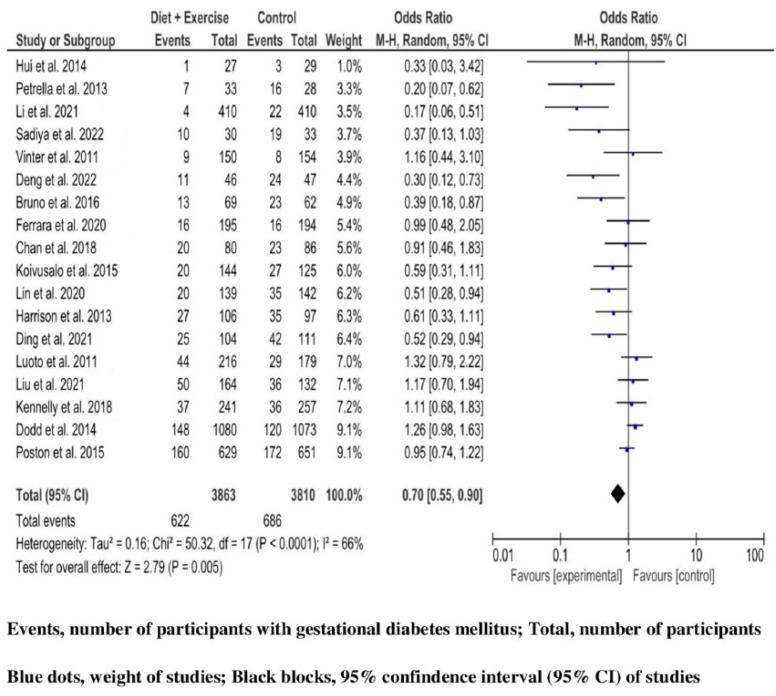
Diet-plus-exercise intervention and the risk of gestational diabetes mellitus in high-risk pregnant women. Metrics of RCT in odds ratio (OR) with 95% confidence interval (95% CI)-black blocks. Performance of meta-analysis with random effect model. Studies are presented in ascending order by weight-blue dots [5,6,7,8,10,11,13,17,18,59,60,61,62,63,64,65,66,67].

Finally, among 7673 (3863 in the experimental group, and 3810 in the comparator group) high-risk pregnant women analyzed for GDM undergoing both diet and exercise intervention, 1308 cases (17%) were diagnosed with diabetes of pregnancy (622 (16.1%) in the intervention group, and 686 (18%) in the control group). The combined studies were analyzed by an MA with RE in the presence of significant heterogeneity (Q 50.32, *p*-value < 0.0001, and large variability (I^2^ 66%, (95% CI 44, 79%)). Women in the group of mixed lifestyle intervention had a significant reduction in GDM incidence (OR 0.70, 95%CI 0.55, 0.90; *p*-value 0.005). (Figure 4).

The summary OR remained significant when separate analyses were limited to studies including (a) performance in China (OR 0.55, 95%CI 0.33, 0.90; *p*-value 0.02), and in Italy (OR 0.32, 95%CI 0.16, 0.60; *p*-value 0.0005); (b) to studies with more than 10% of participants having a low education level (OR 0.73, 95%CI 0.55, 0.95; *p*-value 0.02); (c) to trials without considering increased BMI as a GDM risk factor (OR 0.17, 95%CI 0.06, 0.51; *p*-value 0.001); and (d) to trials with interventions lasting less than 20 weeks (OR 0.81, 95%CI 0.67, 0.97; *p*-value 0.02). The test of difference was significant for subgroup analyses based on the countries where studies were performed (*p*-value 0.02), based on increased BMI as a GDM risk factor (*p*-value 0.003), and based on intervention duration (*p*-value 0.03). In contrast, the test of difference for subgroup analyses based on participant’s low education level was non-significant (*p*-value 0.34) (Table S10). 

Based on the sensitivity analyses, the summary OR remained statistically significant when evaluating the effect of the RCT with the largest sample size [49] (OR 0.67, 95%CI 0.52, 0.86; *p*-value 0.002), and the RCT with a high attrition bias (drop-out ratio 31.4%) [62] (OR 0.73, 95%CI 0.57, 0.93; *p*-value 0.01) (Table S10). The meta-regression analyses with covariates of baseline risk and study duration did not show an effect on the summary OR (Table S11).

### 4.5. Quality of Reporting, Potential Bias, and Quality of Evidence

Assessing the quality of RCTs with dietary intervention, some of them were considered dubious according to specific quality reports. Particularly, three trials did not provide information on blinding of either participants or personnel [3,14,41]; in addition, one of them did not declare allocation concealment [3]. Also, another three trials did not mention participant blinding [40,42,43]. Four studies were considered high-risk regarding blinding of participants and personnel [4,20,44,45]; specifically, three of them were unblinded for participants [4,20,44], and one of them was unblinded for personnel [45] (Table 10).

**Figure 5:**
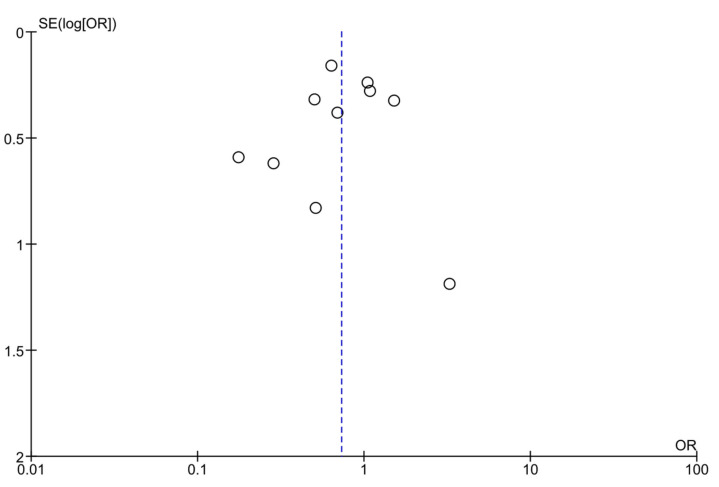
Funnel plot including all studies comparing diet intervention vs. standard prenatal care for gestational diabetes prevention among high-risk pregnant women.

Based on the funnel plot assessment via visualization, the studies did not have a close distribution around the summary effect estimate [38] (Figure 5). Egger’s test of small study effects had a *p*-value of 0.726.

There was potential performance bias in four out of ten RCTs, and it was rated unclear in the remaining six. The overall quality of evidence was moderate for dietary interventions during pregnancy compared with standard prenatal care for preventing GDM in high-risk pregnant women. (Table S12).

Evaluating the quality of RCTs with exercise intervention, some concerns were also evident in relation to performance bias. Three studies did not report blinding of either participants, or personnel [49,52,54]. Furthermore, performance bias was considered unclear in five more RCTs [50,51,53,55,56]: three of them did not report information on participant blinding [50,51,53], and two of them did not report on personnel blinding [55,56]. A detection bias could not be excluded in two trials with unclear performance bias [54,56], as information on recording data were not provided. Performance bias was judged as high in five studies [46,47,48,57,62]: one study was unblinded for both participants and personnel [62], and four were unblinded for participants only [30,47,48,57]. In addition, one trial reported a participant drop-out rate of 41% [48] (Table 11).

**Figure 6:**
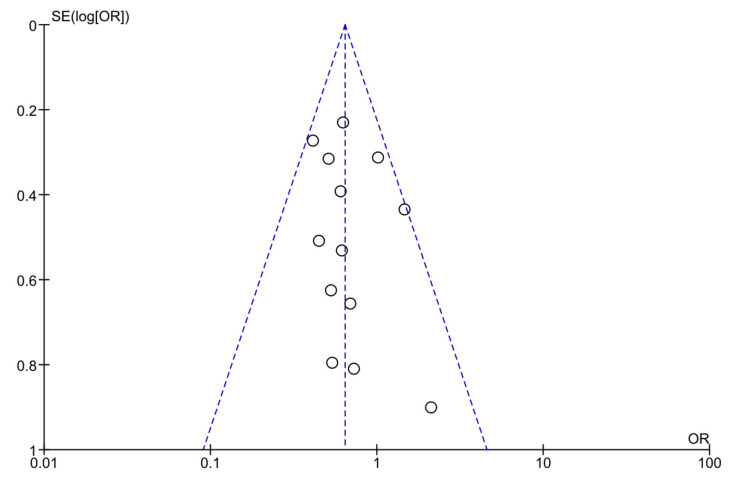
Funnel plot including all studies comparing exercise intervention vs. standard prenatal care for gestational diabetes prevention among high-risk pregnant women.

When examining the funnel plot, variation in the standard error of the studies with exercise intervention is evident. Small studies are positioned closer to the summary effect estimate [38] (Figure 6). However, Egger’s test of small study effects had a *p*-value of 0.399.

In total, five out of thirteen RCTs had potentional performance, or attrition bias. The other eight RCTs were unclear in relation to blinding. The overall quality of evidence was moderate for exercise interventions during pregnancy compared with standard prenatal care for preventing GDM in high-risk pregnant women. (Table S13).

Similar to the quality assessment of RCTs with only dietary, and only exercise intervention, some uncertainty was arose for RCTs with a combined intervention of diet and exercise. Performance bias was assessed as unclear in half of them; in particular, three trials did not give information on either participant or personnel blinding [18,59,64], and six trials only on participant blinding [6,11,58,61,65,66]. In addition, one RCT had a participant drop-out rate of 31.4% [55]. The performance bias was judged as high in the other half of the studies; specifically, seven were unblinded for participants and personnel [5,7,8,17,60,63,67], and two trials were unblinded for participants [10,13] (Table 12).

**Figure 7:**
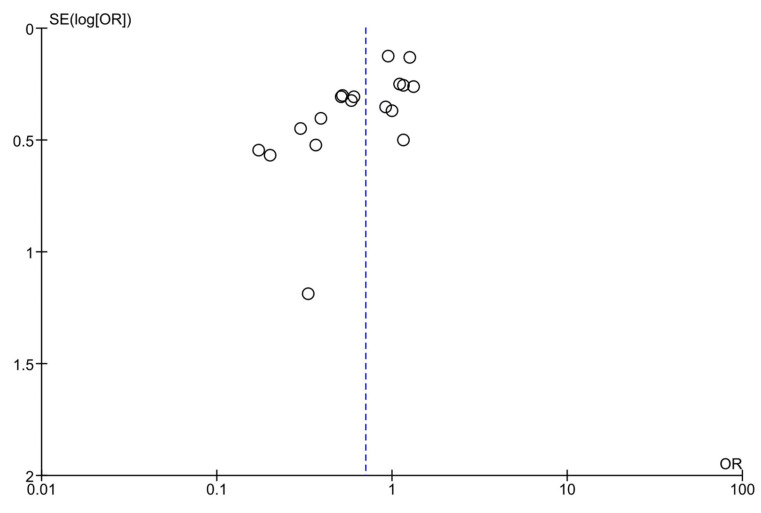
Funnel plot including all studies comparing diet-plus-exercise intervention vs. standard prenatal care for gestational diabetes prevention among high-risk pregnant women.

Studies with exercise intervention form an asymmetry when the funnel plot is visually evaluated [38] (Figure 7). Similarly, Egger’s test of small study effects had a *p*-value of 0.001.

In summary, ten out of eighteen RCTs had a potential performance or attrition bias, and nine were unclear about blinding. The overall quality of evidence was low for dietary plus exercise interventions during pregnancy compared with standard prenatal care for preventing GDM in high-risk pregnant women. (Table S14).

## 5. Discussion

For pregnant women at high risk of GDM, undergoing lifestyle interventions during pregnancy may have benefit on preventing GDM. PA interventions in pregnancy were significant in reducing the incidence of GDM in high-risk women. Combined interventions of diet plus exercise during pregnancy in high-risk pregnant women may also lead to reduced GDM occurrence. However, the protective effect of dietary interventions on GDM was marginally non-significant when they were not combined with exercise during pregnancy in high-risk women. In the subgroup analyses, it was found that the PA interventions also produced significant results for GDM prevention when separate analyses where limited to studies that were conducted in Spain, and in China, to studies with more than 10% of participants having a low education level, independently of overweight or obesity as a GDM risk factor, of intervention duration more than 20 weeks or of emotional support. The effect of a mixed intervention approach on preventing GDM also remained significant in subgroup analyses based on studies that were performed in China and in Italy, in studies involving a percentage of women with low education level more than 10%, independently of increased BMI as a GDM risk factor or duration of intervention below 20 weeks. Dietary interventions in pregnant women at high-risk for GDM that were conducted in Great Britain, and Spain, regardless of participants being overweight or obese or interventions including a Mediterranean diet or motivation arm may have benefits in preventing GDM. Heterogeneity across studies was significant.

Pregnant women, regardless of the risk for GDM, did not benefit from lifestyle or pharmacological interventions during pregnancy in terms of GDM prevention, according to an overview SR [27]. In this study we focused on high-risk pregnant women, assessing the effect of lifestyle interventions on preventing GDM. Previous studies evaluating the preventive role of lifestyle interventions in pregnancy on preventing GDM in high-risk women demonstrated contradictory results. An SR and MA evaluating dietary interventions for preventing GDM reported a possible effect in obese pregnant women [68], and marginally non-significant results in general population [68]. According to three SRs and MAs, overweight and obese pregnant women may also have benefits in prevention of GDM from PA interventions during pregnancy [69,70,71]. Moreover, evaluating overall high-risk GDM pregnant women, independently of obesity, two MAs reported significant results for preventing GDM with exercise during pregnancy [12,17]. However, three SRs and MAs evaluating lifestyle interventions during pregnancy, including diet and PA interventions, among overweight and obese pregnant women found no prevention of GDM [18,28,29]. In another study, the SR and MA did not demonstrate a significant reduction in GDM with PA during pregnancy in overweight and obese women [14]. In this trial, evaluating the effect of lifestyle interventions on preventing GDM in high-risk women with any identified GDM risk factor, we expanded high-risk population under examination. As far as is known, this is the first study appraising systematically the effect of lifestyle interventions during pregnancy on preventing GDM among pregnant women with any risk factor for GDM. 

An SR identified intrapersonal themes as the most frequently reported barriers and enablers to PA during pregnancy [72]. Moreover, obese women were less active compared with normal weight women [53], and their adherence to exercise programs was low [53]. Overweight and obese pregnant women changed their lifestyle habits with difficulty, probably due to a lack of motivation [17], or believing they were not at risk [11]. Furthermore, the efficacy of exercise interventions during pregnancy addressing this population was not often measured [53]. The optimal amount of PA in obesity is unknown, although most guidelines suggest exercising through obese pregnancy [53]. The lack of success of some RCTs in preventing GDM could be attributed to the lack of statistical power and poor adherence to study protocols [73]. In addition, the main issue of performing RCTs with lifestyle interventions concerns performance bias, as blinding either participants or personnel might be difficult due to the nature of the interventions [5,17]. 

Pre-pregnancy increased BMI is a modified and important GDM risk factor [74]. The preconception stage is the critical time for implementing public health strategies attempting to reserve overweight and obesity [74]. Women of childbearing age have been highlighted as a target group for prevention of obesity, according to the WHO [75]. These strategies may target a limitation to gained weight in pregnancy and postpartum [75]. The pre-pregnancy time period would be ideal regarding the implementation of interventions aiming at weight loss [28]. However, such interventions are difficult to achieve because many pregnancies are unplanned (i.e., 50% of pregnancies in the UK), and only a small percentage of women planning pregnancy follow nutrition and lifestyle recommendations [28]. Thus, a pre-pregnancy intervention may reach only a small proportion of the targeted group of women [28].

The antenatal period is considered an ideal time to intervene as mothers are motivated to make changes that could optimise their outcome and that of the baby, with opportunities for regular contact with health care professionals [76]. Trials of lifestyle interventions demonstrating safety and efficacy for both the mother and her offspring, could be performed before gestational metabolic changes during the first trimester of pregnancy [73]. Social support also may have an enabling role [72]. Person-centred strategies using behaviour change techniques should be used to address intrapersonal and social factors to translate pregnant women’s positive attitudes into increased PA participation [72]. Targeted and well-designed exercise programs are needed for obese pregnant women [53]. Furthermore, studies on bariatric surgery prior to pregnancy suggest that interventions targeting maternal obesity can also improve short- and long-term offspring health [57]. 

GDM increases short-term and long-term maternal and offsprings’ complications [7,8]. Major unfavorable short-term conditions include macrosomia [8], pre-eclampsia, [28], preterm birth [8], and polyhydramnios [8]. Furthermore, GDM is a well-established predictor of future DM [6]. Women with GDM, as well as the next generations, have an increased risk of developing Type 2 (T2) DM [16,59]. Particularly, up to 70% of women with GDM may develop T2DM in later life [17]. T2DM is diagnosed soon after delivery in up to 10% of women with previous GDM [6], and its prevalence may approach 38% within the first postpartum year [7]. The percentage of T2DM has been observed to increase by up to 70% in a 10-year duration of follow-up [6], and by up to 60% in the 16th postpartum year [7]. Additionally, women who experience GDM may have an increased risk of developing cancer during their lifetime [2]. GDM pregnancies may increase the overall risk of breast cancer (BC), and especially oestrogen receptor-positive BC [77]. GDM has also been corelated with pancreatic cancer in pregnancy and in later life [78]. 

GDM represents a heavy economic burden on patients and societies due to its diagnosis and treatment [8]. In China, it has been found that GDM diagnosis and treatment, delivery, and the management of a mother’s neonatal complications in the last trimester of pregnancy cost USD 1929.87 on average per woman more than a non-GDM pregnant woman [8]. In Australia, the cost for women with GDM not needing insulin is AUD 2026, AUD 2534 for women needing insulin, and AUD 3826 if fetal heart rate is being monitored via cardiotocography [79]. Also in Australia, according to previous estimations, AUD 53,985 additional direct costs are incurred for every 100 women with a singleton pregnancy and receiving treatment for mild GDM in addition to routine obstetric care, while AUD 6521 additional charges are incurred by women and their families; in addition, AUD 27,503 was the incremental cost per additional serious perinatal complication prevented, AUD 60,506 was the cost per perinatal death prevented, and AUD 2988 was the cost of each discounted life-year gained [80].

Preventing GDM could have greater economic and health benefits than treatment [1]. It is likely that additional health service and personal financial spending on GDM prevention in the general population of high-income countries (HIC) would lead to reductions in perinatal mortality and in serious perinatal complications sufficient to justify the spending [80]. Lifestyle interventions aiming to prevent excessive GWG and GDM in the general population were found to be neutral and possibly cost-effective for the health care system of Australia [81]. The cost-effectiveness evaluation of the multicenter RCT conducted in Europe including diet only or exercise only or a combined approach for pregnant women at increased risk of GDM found that promotion of healthy eating and PA was the preferred strategy for limiting GWG, and for quality of life (QUALY) after delivery; however, the mixed approach of lifestyle interventions was not cost-effective for fasting blood glucose (FBG) [82]. Another assessment of the cost efficacy of a lifestyle intervention for preventing GDM in high-risk pregnant women in Finland showed that interventions targeting neonatal birth weight were not cost-effective [83]. In addition, an analysis of the cost of exercise intervention in high-risk pregnant women in Holland reported no significant results for GDM prevention, FBG, insulin sensitivity, infant birth weight and maternal QUALY [84]. 

Community and personal-based strategies are needed to support social and psychological challenges that pregnant women experience with physical exercise. The time during pregnancy at which lifestyle interventions are initiated varies. However, the majority of eligible RCTs initiated lifestyle interventions before the 20th gestational week. Additionally, the lack of estimation of overall activity level and resting time of participating pregnant women in the RCTs may contribute to conflicting results. The RCTs do not report total physical activity or the resting time of the pregnant women. Potentially, lifestyle variations including the type and time of occupation and the associated leisure time of participant pregnant women may contribute to differences in the efficacy of lifestyle interventions in preventing GDM that are observed across countries.

This study has several limitations. The results cannot be generalized to the general population. In addition to overweight or obesity, women with any risk factor for GDM including ethnicity, medical and family history, and sedentary lifestyle were evaluated, aiming to broaden the criteria of the population under assessment. Therefore, the risk for GDM was not similar. Furthermore, this study included only trials that evaluated lifestyle interventions initiated during pregnancy; therefore, their findings cannot be generalized to lifestyle interventions that may begin in the preconception period. Heterogeneity across studies could not be excluded. Procedures for blinding personnel and/or participants may face some limitations due to the nature of interventions. 

## 6. Conclusions

The findings of this study support the effectiveness of lifestyle interventions during pregnancy, including a separate exercise arm or exercise combined with diet, compared with usual prenatal care, for preventing GDM in high-risk pregnant women. Nutrition interventions alone were marginally insufficient in preventing GDM. However, dietary interventions during pregnancy in the form of Mediterranean dietary patterns that are combined with exercise and are motivationally enhanced may be the preferred strategy for preventing GDM among high-risk women. Future, large, high-quality RTCs and SRs are necessary to confirm the protective effect of lifestyle interventions for GDM. 

## Figures and Tables

**Table 1:** Characteristics of eligible trials with dietary intervention.

Name of First Author, Year of Publication	Country	Number of Participating Centers	Study Design	Study Duration (mo)	Drop-Out Rate *n* (%)
Korpi-Hyövälti, 2011 [40]	Finland	1	Parallel	ND	6 (10.0)
Quinlivan, 2011 [43]	Australia	1	Parallel	ND	8 (6.1)
Walsh, 2012 [41]	Ireland	1	Parallel	48	41 (5.1)
McCarthy, 2016 [42]	Australia	1	Parallel	20	11 (2.9)
Yi Zhang, 2019 [45]	China	2	Parallel	40	31 (10.2)
Al Wattar, 2019 [20]	UK	5	Parallel	17	67 (5.3)
Okesene-Gafa, 2019 [44]	New Zealand	1	2 × 2 Factorial	26	6 (2.6)
Melero, 2020 [3]	Spain	1	Parallel	12	25 (8.8)
Basu, 2021 [4]	USA	1	Parallel	23	11 (24.4)
Dong-Yao Zhang, 2022 [16]	China	1	Parallel	10	6 (5.8)

ND, no data; UK, United Kingdom; USA, United States of America.

**Table 2:** Characteristics of eligible trials with exercise intervention.

Name of First Author, Year of Publication	Country	Number of Participating Centers	Study Duration (mo)	Drop-Out Rate *n* (%)
Do Nascimento, 2011 [52]	Brazil	1	19	2 (2.4)
Oostdam, 2012 [51]	Netherlands	5	48	22 (18.2)
Price, 2012 [54]	USA	1	45	29 (31.9)
Barakat, 2013 [46]	Spain	1	40	82 (16)
Ruiz, 2013 [49]	Spain	3	40	138 (14.3)
Nobles, 2015 [55]	USA	1	60	39 (13.4)
Bisson, 2015 [53]	Canada	1	25	5 (10.0)
Seneviratne, 2015 [57]	New Zealand	1	19	1 (1.3)
Perales, 2016 [48]	Spain	1	49	99 (41.1)
Krohn Garnæs, 2016 [50]	Norway	1	22	17 (18.7)
Guelfi, 2016 [56]	Australia	1	37	3 (1.7)
Wang, 2017 [58]	China	1	20	35 (11.7)
Daly, 2017 [47]	Ireland	1	41	2 (2.3)

USA, United States of America.

**Table 3:** Characteristics of eligible trials with dietary plus exercise intervention.

Name of First Author, Year of Publication	Country	Number of Participating Centers	Study Design	Study Duration (mo)	Drop-Out Rate *n* (%)
Luoto, 2011 [5]	Finland	14	Cluster	14	43 (9.7)
Vinter, 2011 [59]	Denmark	2	Parallel	36	56 (15.5)
Harrison, 2013 [11]	Australia	3	Parallel	ND	25 (10.1)
Petrella, 2013 [61]	Italy	1	Parallel	6	0 (0)
Dodd, 2014 [65]	Australia	3	Parallel	30	70 (3.2)
Hui, 2014 [67]	Canada	1	Parallel	28	0 (0)
Poston, 2015 [60]	UK	8	Parallel	62	275 (17.7)
Koivusalo, 2015 [6]	Finland	4	Parallel	71	24 (8.2)
Bruno, 2016 [62]	Italy	1	Parallel	16	60 (31.4)
Kennelly, 2018 [17]	Ireland	1	Parallel	35	67 (11.8)
Chan, 2018 [13]	China	1	Parallel	24	63 (27.5)
Ferrara, 2020 [66]	USA	5	Parallel	42	4 (1)
Lin, 2020 [18]	China	1	Parallel	5	23 (7.6)
Li, 2021 [64]	China	1	Parallel	10	ND
Liu, 2021 [63]	China	1	Parallel	27	58 (15.1)
Ding, 2021 [7]	China	1	Parallel	10	15 (6.5)
Deng, 2022 [8]	China	1	Parallel	11	10 (10.6)
Sadiya, 2022 [10]	UAE	1	Parallel	22	7 (11.1)

ND, no data; UK, United Kingdom; USA, United States of America; UAE, United Arab Emirates.

**Table 4:** Characteristics of participants in studies with dietary intervention.

Name of First Author, Year of Publication	Sample Size (Intervention/Control)	Mean Age (SD), yr Intervention/Control	Low Education Level, *n* (%) Intervention/Control	Risk Factors for GDM
Korpi-Hyövälti, 2011 [40]	60 (30/30)	ND	ND	At least one of (1) advanced maternal age (>40 years), (2) overweight or obesity (BMI > 25), (3) family history of DM, (4) history of GDM or history of macrosomia
Quinlivan, 2011 [43]	132 (67/65)	28.3/29.5	ND	Overweight (BMI 25–29.9) or obesity (BMI > 29.9)
Walsh, 2012 [41]	800 (394/406)	32.0 (4.2)/32.0 (4.2)	ND	History of macrosomia
McCarthy, 2016 [42]	382 (190/192)	31.9 (4.6)/31.8 (4.6)	20 (10.5)/15 (7.8)	Overweight or obesity (BMI ≥ 25)
Yi Zhang,2019 [45]	400 (200/200)	28.1 (3.6)/28.0 (3.7)	ND	Overweight or obesity (BMI ≥ 24)
Al Wattar, 2019 [20]	1252 (627/625)	31.4 (5.2)/30.9 (5.2)	ND	At least one of (1) obesity (BMI ≥ 30), (2) raised serum triglycerides (≥1.7 mmol/L), (3) chronic HY (systolic blood pressure ≥ 140 mm Hg or diastolic blood pressure ≥ 90 mm Hg)
Okesene-Gafa, 2019 [44]	230 (116/114)	29.8 (5.7)/27.8 (5.5)	35 (30.2)/34 (29.8)	Obesity (BMI ≥ 30)
Melero, 2020 [3]	285 (143/142)	31.7 (5.4)/31.3 (5.6)	18 (12.6)/28 (19.7)	Hispanic origin
Basu, 2021 [4]	45 (22/23)	27.0 (5.3)/27.0 (5.0)	ND	Obesity (BMI ≥ 30) AND at least one of (1) family history of DM, (2) history of GDM
Dong-Yao Zhang, 2022 [16]	104 (52/52)	31.1 (4.2)/30.0 (4.0)	ND	Overweight or obesity (BMI ≥ 24)

ND, no data; BMI, body mass index, DM; diabetes mellitus; GDM, gestational diabetes mellitus; HY, hypertension.

**Table 5:** Characteristics of participants in studies with exercise intervention.

Name of First Author, Year of Publication	Sample Size (Intervention/Control)	Mean Age (SD), yr Intervention/Control	Low Education Level, *n* (%) Intervention/Control	Risk Factors for GDM
Do Nascimento, 2011 [52]	82 (40/42)	29.7 (6.8)/30.9 (5.9)	6 (15)/10 (23.8)	Overweight (BMI 26.0–29.9) or obesity (BMI ≥ 30.0)
Oostdam, 2012 [51]	121 (62/59)	30.1 (4.5)/30.8 (5.2)	16 (34)/17 (34.7)	Overweight (BMI ≥ 25) or obesity (BMI ≥ 30.0) AND at least one of (1) family history of DM, (2) history of GDM, (3) history of macrosomia
Price, 2012 [54]	91 (43/48)	30.5 (5)/27.6 (7.3)	ND	Sedentary lifestyle: no aerobic exercise more than once per week for at least the past six months
Barakat, 2013 [46]	510 (255/255)	31 (3)/31 (4)	54 (24.7)/75 (34.4)	Sedentary lifestyle: not exercising more than 20 min on more than 3 days/week
Ruiz, 2013 [49]	962 (481/481)	31.6 (6.4)/31.9 (4)	211 (43.9)/183 (38.3) *	Sedentary lifestyle: not exercising more than 20 min on more than three days/week
Nobles, 2015 [55]	290 (143/147)	Range 18–40	26 (22.2)/31 (27.4)	One of overweight or obesity (BMI ≥ 25) AND family history of DM, (2) history of GDM
Bisson, 2015 [53]	50 (25/25)	30.5 (3.7)/31.0 (4.0)	10 (40)/9 (36)	Obesity (BMI ≥ 30.0)
Seneviratne, 2015 [57]	75 (38/37)	ND	ND	Overweight or obesity (BMI ≥ 25)
Perales, 2016 [48]	241 (120/121)	31 (4)/31 (4)	29 (24)/30 (25)	Sedentary lifestyle: not exercising regularly more than 30 min on three days/week
Krohn Garnæs, 2016 [50]	91 (46/45)	31.3 (3.8)/31.4 (4.7)	1 (2.3)/3 (7.0)	Overweight or obesity (BMI ≥ 28.0)
Guelfi, 2016 [56]	172 (85/87)	33.6 (4.1)/33.8 (3.9)	ND	History of GDM
Wang, 2017 [58]	300 (150/150)	32.1 (4.6)/32.5 (4.9)	31 (20.7)/40 (26.7)	Overweight or obesity (BMI ≥ 24.0)
Daly, 2017 [47]	88 (44/44)	30.0 (5.1)/29.4 (4.8)	ND	Obesity (BMI ≥ 30.0)

ND, no data; BMI, body mass index; DM, diabetes mellitus; GDM, gestational diabetes mellitus; min, minutes; * no university education.

**Table 6:** Characteristics of participants in studies with dietary plus exercise intervention.

Name of First Author, Year of Publication	Sample Size (Intervention/Control)	Mean Age (SD), yr Intervention/Control	Low Education Level, *n* (%) Intervention/Control	Risk Factors for GDM
Luoto, 2011 [5]	442 (246/196)	29.5 (4.8)/30.0 (4.7)	73 (33.8)/59 (33.7)	At least one of (1) advanced maternal age (≥40 years), (2) overweight or obesity (BMI ≥ 25), (3) family history of DM, (4) history of GDM or history of macrosomia
Vinter, 2011 [59]	360 (180/180)	29 (Range 27–32)/29 (Range 26–31)	39 (26.0)/54 (35.0)	Obesity (BMI 30.0–45)
Harrison, 2013 [11]	228 (121/107)	32.4 (4.6)/31.7 (4.5)	20 (16)/12 (11)	Overweight (BMI ≥ 25 or ≥23 if high-risk ethnicity (Polynesian, Asian, African) or obesity (BMI ≥ 30.0)
Petrella, 2013 [61]	61 (33/28)	31.5 (4.2)/32.4 (5.9)	11 (33.3)/13 (43.3)	Overweight or obesity (BMI ≥ 25)
Dodd, 2014 [65]	2202 (1105/1097)	29.3 (5.4)/29.6 (5.6)	ND	Overweight or obesity (BMI ≥ 25)
Hui, 2014 [67]	113 (57/56)	31.0 (4.0)/32.0 (5.0)	ND	Overweight or obesity (BMI ≥ 25)
Poston, 2015 [60]	1555 (783/772)	30.5 (5.5)/30.4 (5.6)	ND	Obesity (BMI ≥ 30.0)
Koivusalo, 2015 [6]	293 (155/138)	32.3 (4.9)/32.6 (4.5)	4 (3)/3 (2)	At least one of (1) obesity (BMI ≥ 30.0), (2) history of GDM
Bruno, 2016 [62]	191 (96/95)	31.5 (5.0)/30.8 (5.5)	39 (40.6)/35 (35.9)	Overweight or obesity (BMI ≥ 25)
Kennelly, 2018 [17]	565 (278/287)	32.8 (4.6)/32.1 (4.2)	7 (2.7)/6 (2.2)	Overweight or obesity (BMI 25.0–39.9)
Chan, 2018 [13]	229 (118/111)	33.2 (4.4)/33.1 (4.1)	31 (38.8)/41 (47.7)	At least one of (1) advanced maternal age (≥35 years), (2) overweight or obesity (BMI ≥ 25), (3) family history of DM, (4) history of GDM or history of macrosomia
Ferrara, 2020 [66]	398 (200/198)	32.4 (4.1)/32.6 (4.3)	10 (5)/12 (6)	Overweight or obesity (BMI 25.0–40.0)
Lin, 2020 [18]	304 (152/152)	31.4 (4.9)/31.8 (5.1)	ND	At least one of (1) advanced maternal age (≥35 years), (2) overweight or obesity (BMI ≥ 25), (3) family history of DM, (4) history of GDM (5) history of PCOS
Li, 2021 [64]	820 (410/410)	37.5 (3.5)/38 (2.5)	ND	Older pregnant women
Liu, 2021 [63]	384 (192/192)	31.8 (3.4)/32.3 (3.8)	ND	Overweight or obesity (BMI ≥ 25) AND history of PCOS
Ding, 2021 [7]	230 (114/116)	30.6 (2.8)/30.1 (2.7)	ND	Overweight or obesity (BMI ≥ 24)
Deng, 2022 [8]	94 (47/47)	29.6 (3.9)/29.6 (3.4)	ND	At least one of (1) advanced maternal age (≥35 years), (2) overweight or obesity, (3) family history of DM, (4) history of GDM, (5) history of macrosomia, (6) history of PCOS, (7) history of abnormal lipid metabolism, (8) elevated FPG in early pregnancy (FPG ≥ 5.1 mmol/L)
Sadiya, 2022 [10]	63 (30/33)	32.8 (4.1)/30.8 (5.2)	3 (10)/6 (18)	High-risk ethnic group (Middle Eastern, Southern Asian) AND at least two of (1) obesity (BMI ≥ 30), (2) family history of DM, (3) history of GDM, (4) history of macrosomia, (5) history of PCOS

ND, no data; BMI, body mass index; DM, diabetes mellitus; GDM, gestational diabetes mellitus; PCOS, polycystic ovary syndrome; FPG, fasting plasma glucose.

**Table 7:** Eligible studies with dietary intervention.

Name of First Author, Year of Publication	Provider	Duration of Intervention	Description of Dietary Intervention	Side Effects/Adverse Events (Intervention/Control)
Korpi-Hyövälti, 2011 [40]	Clinical nutritionist	From GW 8–12 until GW 36–40	Delivery: Individual dietary advice, four times in the first and second trimesters, and two times in the third trimester. Components: Food record before the first appointment at GW 8–12. Repetition of food record procedure before fifth and sixth appointment at GW 26–28, and 36–40, respectively. Nutrition rich in fruits, vegetables, and berries, consumption of fat-free and low-fat dairy products, vegetable oils, and wholegrain products. Daily energy intake of 126 kJ/kg and 105 kJ/kg for normal-weight, and for overweight women, respectively, divided into carbohydrate 50–55 E%, fat 30 E%, saturated fat < 10 E%, protein 15–20 E%, and dietary fiber at least 15 g/4184 kJ (1000 kcal). Motivation: Informative. Assessment: Questionnaires, nutrient calculation software, food records analyses.	ND
Quinlivan, 2011 [43]	Food technologist	ND	Delivery: Dietary intervention at antenatal visits with weighing, and continuous care provider. Components: Increasing consumption of fresh vegetables, fresh fruit, home-cooked main meal, and water; decreasing consumption of carbonated drinks, sports drinks, commercial fruit juices, and fresh fast foods. Motivation: Psychological assessment and intervention if necessary. Assessment: ND	Preterm delivery (1/1), acute polyhydramnios (0/1)
Walsh, 2012 [41]	Dietitian	From GW 15 ± 3 until GW 34	Delivery: Dietary sessions in a group of two to six women, lasting 2 h. Components: Firstly, general advice on guidelines of healthy eating during pregnancy. Recommended low-GI eucaloric diet, and not reducing total caloric intake. Focus of education sessions on the GI. Encouragement to choose many low-GI foods instead high-GI foods. Motivation: Answers to any dietary queries of participants, written information regarding low-GI nutrition. Assessment: Food diaries for estimation of the GI, questionnaires for assessment of adherence to the low-GI diet.	Stillbirths (1/0)
McCarthy, 2016 [42]	Midwives	From GW < 20 until GW 36	Delivery: Individual sessions provided by research midwife of approximately 30 min at subsequent antenatal appointments. Components: Nutrition advice, encouragement to record weight, and discussion of weight gain with doctors and/or midwives. Motivation: N/a Assessment: Self-weight record in light indoor clothing, questionnaires	Early pregnancy loss (2/1)
Yi Zhang,2019 [45]	Dietitians	From GW ≤ 16 until GW 34–36	Delivery: Individualized dietary consultation by separate dietitians on a different day. Components: Individualized diet planning for achievement of a low-GI goal with consideration of individual food preference. Participants nutrition assessment through 24 h food recall intake of the nearest working day. Also, diet GI and GL calculations in the diet assessment. Equipment of a mobile phone app DietGI with the function of calculating GI and GL for every selected food. Motivation: Counseling about substituting high-GI with low-GI foods, cooking techniques, and combining foods in meals. Assessment: Worksheet for diet records, and customized excel worksheet for calculation of GI and GL.	Miscarriage (5/4)
Al Wattar, 2019 [20]	Dietitian, trained researchers	From GW 18 until GW 28	Delivery: Three sessions: a personalised session at GW 18, and two sessions in a group at GW 20 and 28, using presentations. In addition, permission for presence women’s spouses. Components: Firstly, 24 h food recall and consumption needed to follow a Mediterranean pattern of diet. A book providing cooking advice on Mediterranean diet. Diet with increased consumption of fruit, vegetables, non-refined grains, legumes, nuts, and EVOO; moderate to high fish intake; low to moderate poultry and dairy products consumption; low red and processed meat intake; and avoidance of sugary drinks, fast food, and animal fat. Provision of mixed nuts and EVOO as main sources of fat. Motivation: Follow-up telephone calls Assessment: Number of attended sessions, questionnaires	None reported
Okesene-Gafa, 2019 [44]	Dietitian, community health workers	From GW 12^+0^–17^+6^ until delivery	Delivery: Four education sessions at home. Development of a manual provided by a dietitian. Components: A HUMBA handbook with advice on healthy nutritious foods, unhealthy drinks, and recipes. Behavioural change techniques promoting healthy diet, and setting SMARTER goals. Motivation: Feedback, positive reinforcement for goals achieved, motivational text messages Assessment: A personal pregnancy weight-gain chart, number of attended sessions, questionnaires.	Fetal death (1/3)
Melero, 2020 [3]	Dietitian	From GW 8–12 until delivery	Delivery: Four clinic-based visits of participants. Components: Counseling on Mediterranean diet, and increased consumption of EVOO and nuts; daily consumption of EVOO ≥ 40 mL, and pistachios 25–30 g for at least 3 days weekly. Free provision of 10 L of EVOO, and 2 kg of pistachios during the first and second visit. Advice against consumption of alcohol and juices. Motivation: Free of cost supplies Assessment: Questionnaires, MEDAS for assessment adoption of Mediterranean Diet; MEDAS score 0–12 points, without consideration of alcohol and juice.	ND
Basu, 2021 [4]	Dietitian, nurse practitioner	From GW < 20 until GW 32–36	Delivery: Three clinic-based visits of participants: one at baseline, GW < 20, and two subsequent visits in the second, and third trimesters at GW 24–28 and 32–36, respectively. Components: Maintenance of habitual diet throughout the study (280 g daily food intake of 160 kcal divided into 38 g total carbohydrates, 8 g total fiber, 8 mg vit C, 3 mg sodium, 168 mg K, 1600 mg total polyphenols, and 700 mg anthocyanins). Consumption of two cups of frozen blueberries in mid-morning, or afternoon, or evening as a snack by itself and not in combination with any other food items. Intake of 12 g soluble fiber daily from meals. No consumption of fruit juice. Motivation: Free of cost food supplies, education, telephone calls Assessment: Return of any unconsumed supplementation, 24 h food recalls, nutrition calculation software.	Miscarriage (1/1)
Dong-Yao Zhang, 2022 [16]	Νutritionists	From GW 20^+0^–24^+6^ until delivery	Delivery: Dietary education and advice according the Chinese Dietary Guidelines for pregnancy. Components: Participants recalls of amount and frequency consumption of each food in the last five weeks, at GW 20 and 25. In addition to the above guidelines, consumption of soluble powder of fiber twice daily at the total amount of 12 g (energy, 51.93 kcal; carbohydrates, 3.31 g; fiber, 9.78 g). Motivation: Presentations Assessment: Questionnaires, Chinese Food Composition Table for evaluation of mean daily total energy intake	Dietary fiber intolerance (2/0), loss of pregnancy (0/1)

GW, gestational week; E%, energy percent; kJ, kilo joule; kcal, kilocalories; L, liter; mL, milliliter; kg, kilogram; g, gram; GI, glycaemic index; GL, glycaemic load; min, minute; K, potassium; vit, vitamin; EVOO, extra virgin olive oil; app, application; SMARTER, specific, measurable, action-oriented, realistic, timed, evaluated, and reviewed; HUMBA, Healthy Mums and Babies Study; MEDAS, Mediterranean Diet Adherence Screener; ND, no data; N/a, not applicable; h, hour.

**Table 8:** Eligible studies with exercise interventions.

Name of First Author, Year of Publication	Provider	Duration of Intervention	Description of Exercise Intervention	Side Effects/Adverse Events (Intervention/Control)
Do Nascimento, 2011 [52]	Physiotherapist	From GW 14–24 until GW 36	Delivery: Individualized or in a group, supervised exercise program of weekly classes. Components: PA of light to moderate intensity with HR less than 140 beats per min, according to the ACOG recommendations (2002). Total duration of 40 min: stretching for 10 min, strengthening exercises including lower and upper limb muscles for 22 min, and relaxing for 10 min. In addition, home-based exercise five times weekly, or walking. Motivation: Counseling Assessment: Self-reported exercise journal	None reported
Oostdam, 2012 [51]	Physiotherapist	From GW 15 until delivery	Delivery: Individualized, supervised exercise program. Components: Light intensity beginning with a warm-up period for 5–10 min. Next, program’s core for 40 min consisting of aerobic and strength exercises. Finally, a cool-down period for 5–10 min. Motivation: Information about the benefits of exercise in pregnancy. Assessment: Accelerometer for moderate and vigorous activity, METs with cut-off values from the ACSM statement	None reported
Price, 2012 [54]	Researchers	From GW 12–14 until GW 36 or until delivery if participants wished	Delivery: Both individualized and in a group, supervised exercise program performed four times per week. Components: Aerobic PA of moderate intensity for of 45–60 min, according to the ACOG guidelines. Activities: performance of step aerobics (first day), walking in a group over hilly terrain (second day), training circuit (third day), and individual walking for 30 to 60 min (fourth day). Substitution with weight training for 1–10 min, after performing aerobic exercise with an equal time interval. End of sessions with stretches of lower limb muscles for 5 min. Motivation: N/a Assessment: Questionnaires, RPE on the Borg Scale, produced power during the timed walking, documentation of temperature, sit-and-reach test.	Anxiety with exercise (1/0), history of preterm pregnancy (1/0), pain from leiomyomas (1/0)
Barakat, 2013 [46]	Fitness specialist; obstetrician	From GW 10–12 until GW 38–39	Delivery: In a group of 10–12 women, supervised exercise program for three sessions per week (Monday, Wednesday, and Friday). Components: Aerobics, strength and flexibility exercises for 50–55 min, according to the ACOG guidelines. A gradual warm-up and a cool-down period preceding and following main part, respectively, both for 10–12 min. Main session of 25–30 min, including resistance exercises of moderate intensity. In addition, aerobic dance at one session per week, in sections of three to four min with one min breaks including stretching and relaxation activities. Motivation: Sessions accompanied with music, and performance in an airy, well-lighted exercise room at the Hospital. Assessment: HR monitors during the training sessions, Borg’s Scale.	Premature labour (5/3), pregnancy-induced HY (5/4), persistent bleeding (3/0), molar pregnancy (0/3)
Ruiz, 2013 [49]	Researchers	From GW 9 until GW 38–39	Delivery: In a group of 8–10 women, supervised exercise program of three times a week (Monday, Wednesday, and Friday). Components: Aerobic and unaerobic PA of light to moderate intensity for 50–55 min per session. In the beginning, a warm-up period of light intensity for 10-min. Afterward, main part for 25 to 30 min, including aerobic exercises once a week (usually on Friday), and resistance exercises twice a week (usually on Monday and Wednesday). In the end, a cool-down period of light intensity for 10-min. Motivation: N/a Assessment: HR monitors, RPE on the Borg Scale	Threat of premature delivery (14/11), persistent bleeding (7/9)
Nobles, 2015 [55]	Health educators	10 GW on average	Delivery: N/a both design (individualized or in a group), and guidance. Performance on most days of the week. Components: Self-selected specific activities (i.e., dancing, walking, and yard work) of moderate-intensity, for 30 min at least. Motivation: Telephone calls, emails Assessment: Questionnaires, and responses-based individual manual.	Medical contraindication (3/1), miscarriage or termination (1/2)
Bisson, 2015 [53]	Kinesiologists	From GW 15 until GW 27	Delivery: Individualized, supervised exercise program, three times weekly. Components: Consistent with the ACSM Guidelines, moderate-intensity exercise program, and duration of 1 h per session. Session’s content: a warm-up period on a stationary ergocycle for 5–10 min, treadmill walk for 15–30 min, strength PA for 20 min, and finally, relaxing. Motivation: Pamphlet, kinesiologists always available for counseling, modification of the resistance exercises, counseling Assessment: Number of completed sessions, HR monitors, exercise log, RPE on the Borg Scale, accelerometer, questionnaires, METs	None reported
Seneviratne, 2015 [57]	Exercise physiologist	From GW 20 until GW 35	Delivery: Individualized, unsupervised home-based exercise program. Varied frequency and duration, according to stage of pregnancy, between three and five sessions per week, and 15 and 30 min per session, respectively. Components: Home-based exercise sessions of moderate intensity. A warm-up and a cool-down period of low intensity for 5 min, and stationary cycling as main session’s part of moderate intensity. Motivation: Support by an available exercise physiologist. Assessment: HR monitors	None reported
Perales, 2016 [48]	Fitness instructors	From GW 9–11 until GW 38–39	Delivery: In a group of 8–10 women, supervised exercise program, sessions thrice weekly (Monday, Wednesday, and Friday). Components: Session’s duration of 55–60 min. Same structure of all sessions; in the beginning a warm-up period for 5 to 7 min, next aerobic and resistance PA of moderate intensity for 25–30 min, and finally a cool-down period for 5 to 10 min. Motivation: N/a Assessment: HR monitors, resting HR values.	Risk of preterm delivery (4/5), obstetric complications (3/4)
Krohn Garnæs, 2016 [50]	Physiotherapist	From GW 12–19 until delivery	Delivery: In a group, supervised exercise sessions, thrice weekly. Components: PA program for 60 min; aerobic PA of moderate intensity for 35 min, and strength PA for 25 min. Additionally, home-based exercise program at least once weekly for 50 min; endurance training for 35 min, and strength exercises for 15 min. Motivation: Encouragement, and motivational interview individually or in a group. Assessment: Self-reporting in a training diary, Borg Scale, and individually adjusted resistance training.	None reported
Guelfi, 2016 [56]	Exercise physiologist	From GW 14 until GW 28	Delivery: Individualized, supervised sessions of stationary cycling at home, three times per week. Components: Beginning with a warm up period for 5 min. Main part of session with periods of continuous cycling of moderate intensity for 5 min, and periods of interval cycling for 5 min; two types of intervals: an increase in pedaling rate for 15 sec, and an increase in cycling resistance for 30 sec repeated every 2 min. Finish with a cool-down period for 5 min. Motivation: N/a Assessment: Accelerometer, questionnaires	Pregnancy loss (1/2)
Wang, 2017 [58]	Researchers	From GW < 12^+6^ until GW 36–37 (27 ± 2 weeks)	Delivery: Supervised cycling program, at least three times per week. Components: Progressively increased PA according to individual ability. Exercise of low intensity in the beginning of the intervention, cycling for 30 min. Continuous increase in the duration until 45–60 min by adding 5 min to the cycling phases of moderate intensity or to the intervals of cycling. A warm-up and cool-down period for 5 min of each one, in the beginning, and in the end of sessions, respectively. Motivation: N/a Assessment: Questionnaires, RPE on the Borg Scale, records	Cervical length < 25 mm (1/5), low-lying placenta (1/0), ankle sprain (1/0), malformation (0/1), fetal death in utero (0/1)
Daly, 2017 [47]	Researchers	From GW 13^+4/7^ ± 1^+2/7^ until 6 weeks postpartum	Delivery: In a group, supervised exercise program, on a choice of performance day and h. Components: Exercise program for 50–60 min with a warm-up period for 10 min, aerobic resistance exercise both for 15–20 min, and a cool-down period for 10 min. Motivation: Secret Facebook group, SMART goal-setting, classes in a group, journaling, choice of day and h, variance of classes each day, and assurance of childcare during classes. Assessment: HR monitoring, and Borg Scale, according to the ACOG guidelines.	None reported

GW, gestational week; IOM, International Institute of Medicine; ACOG, American College of Obstetricians and Gynecologists; ACSM, American College of Sports Medicine; GWG, gestational weight gain; SMART, specific, measurable, action-oriented, realistic, timed; PA, physical activity; MET, metabolic equivalent task; VO2, volume of oxygen; RPE, perceived exertion; HY, hypertension; HR, heart ratio; HRR, HR reserve; min, minute; sec, second; ND, no data; N/a, not applicable; h, hour.

**Table 9:** Eligible studies with dietary plus exercise interventions.

First Author, Publication Year	Provider	Duration of Intervention	Description of Diet Intervention	Description of Exercise Intervention	Side Effects/Adverse Events (Intervention/Control)
Luoto, 2011 [5]	Nurses	From GW 8–12 until GW 37	Delivery: Dietary counseling session at four visits. Components: Advice on consuming vegetables, fruits, and berries, preferably at least five portions (400 g) a day; selecting mostly high-fiber bread (>6 g fiber/100 g) and other wholemeal products; selecting mostly fat-free or low-fat versions of milk and milk products and of meat and meat products; eating fish at least twice per week (excluding the fish species not recommended for pregnant women); using moderate amounts of soft table spreads on bread, oil-based salad dressing in salad, and oil in cooking and baking; consuming seldom and only in small portions foods high in fat; and consuming seldom and only in small-portions snacks containing high levels of sugar and/or fat Motivation: Counseling cards Assessment: Participants’ notebooks, questionnaires	Delivery: Monthly thematic exercise program in a group Components: Progressive increase in minimum weekly resting time PA dose at 800 MET min, including light-intensity PA. Maximum of 750 MET min of moderate-intensity PA. Motivation: PA counseling Assessment: Self-reports, questionnaires, METs	Miscarriages (6/8)
Vinter, 2011 [59]	Dietitians, physiotherapists	From GW 10–14 until GW 34–36	Delivery: Dietary counseling on four sessions at GW 15, 20, 28, and 35. Components: Dietary advice based on the official Danish recommendations; participants’ individually estimated energy requirements, according to weight and level of activity. Motivation: Encouragement Assessment: Online computer program	Delivery: In a group, supervised exercise program of 1 h weekly. Components: Aerobic; low-step, resistance; light weights, elastic bands, and balance exercises. In addition, advice for being moderately physically active 30–60 min daily. Motivation: Encouragement, free full-time membership in a fitness center Assessment: Bench platform, pedometer, online computer program	Missed abortion (1/4)
Harrison, 2013 [11]	Health trainer	From GW 14–16 until GW 28	Delivery: Four sessions of participants’ individually determined goals. Components: Reduced consumption of high-fat or convenience foods, and increased consumption of fruit and vegetables Motivation: Lifestyle messages Assessment: Self-monitoring based on IOM recommendations	Delivery: Individualised exercise program. Components: Moderate and vigorous activity of daily walking. Motivation: Lifestyle messages Assessment: Self-monitoring, pedometers, questionnaires, METs	Miscarriages or termination (1/2), premature delivery (3/1)
Petrella, 2013 [61]	Dietitian	From GW 16 until GW 36	Delivery: 1 h counseling session Components:1500 kcal/day, divided into three main meals, and three snacks; due to PA program, an addition of 200 kcal/day for obese, and 300 kcal/day for overweight women. Macronutrient diet target composition of 55% carbohydrates of at least of 225 g/day to prevent ketosis (80% complex low GI, 20% simplex), 20% protein (50% animal, 50% vegetable), and 25% fat (12% mono-unsaturated, 7% polyunsaturated, and 6% saturated) with moderately low saturated fat levels. Motivation: Counseling Assessment: Questionnaires, urine exams (ketonuria)	Delivery: PA program at least thrice a week Components: 30 min of moderate intensity activity Motivation: Exercise and lifestyle surveys Assessment: Pedometers, talk test	ND
Dodd, 2014 [65]	Dietitian, trained research assistants	From GW 10–16 until GW 36	Delivery: A planning session with a research dietitian Components: Nutrition counseling according to Australian standards for a balanced consumption of carbohydrates, fat, and protein. Consumption of two servings of fruit, five servings of vegetables, and three servings of dairy each day, and reduced intake of foods high in refined carbohydrates and saturated fats, while increasing intake of fiber. Motivation: Encouragement, support, individualized facilitators, telephone calls Assessment: Self-monitoring, workbook	Delivery: Generic Components: PA advice primarily on increasing walking and incidental activity. Motivation: Encouragement, support, individualized facilitators, telephone calls Assessment: Self-monitoring, workbook	Stillbirths (5/5), miscarriages (25/25); neonatal deaths (4/1); maternal deaths (1, motor vehicle collision)/(1, ruptured maternal splenic artery aneurysm)
Hui, 2014 [67]	Dietitians	From GW 20–26 until GW 36	Delivery: A personalized, individualized nutrition consultation, with the assistance of FCM interview tool (possibility of receiving a complete weekly intake and decisions for food choices). Components: Nutritional recommendations based on the dietary intake analysis and Health Canada guidelines for food intake in pregnancy with considerations for personal food preference, food beliefs, and food budgeting. Motivation: Food stickers on a magnetic board, nutritional information on the food sticker scanned into the computer, instant analysis of daily calorie intake and macronutrients. Assessment: Self-reported food intake records, software containing Canadian Food Database	Delivery: Group or individualized PA program or at home, 3–5 times weekly. Components: Aerobic exercise, strength exercise, and stretching of mild to moderate intensity, in a group or at home according to an exercise DVD instruction, for 30–45 min. Motivation: DVD, exercise logbook Assessment: Questionnaires	ND
Poston, 2015 [60]	Health trainer	From GW 20^+6/7^ until GW 28^+6/7^	Delivery: Eight health trainer-led group or individual sessions of 1 h duration once a week for 8 weeks. Components: Promotion of a healthy pattern of eating but not necessarily restriction of energy intake. Recommendations tailored to the woman’s habitual diet and cultural preference. Suggested exchanging carbohydrate-rich foods with a medium-to-high GI for those with a lower GI to reduce the GL, and restricting dietary intake of saturated fat. Motivation: Telephone calls, emails, SMART goals, handbook counseling, encouragement Assessment: Self-monitoring, log book, dietary data, GWG, anthropometry, questionnaires, and nutrition calculation software.	Delivery: Eight health trainer-led group or individual sessions of 1 h duration once a week for 8 weeks Components: Walking at a moderate, progressively increased intensity, tailored to women’s pre-existing activities. Additional PA options for previously active women. Motivation: SMART goals, handbook, counseling, encouragement, DVD Assessment: Pedometer, log book, PA scores, GWG, anthropometry, questionnaires, METs	Loss of pregnancy (14/14), miscarriages (6/2), fetal deaths in utero (2/4), termination (1/3)
Koivusalo, 2015 [6]	Dietitians, study nurses	From GW 13.3 (12–14.6) until GW 35.1 (34.4–35.6)	Delivery: In the beginning, counseling session in a group led by a dietitian. After enrollment, three sessions of individualized counseling according to the stage of the pregnancy, led by the study nurses. Components: Dietary advice based on contemporary Nordic Nutrition Recommendations 2004. Focus on optimizing participants’ consumption of vegetables, fruits and berries, wholegrain products rich in fiber, low-fat dairy products, vegetable fats high in unsaturated fatty acids, fish, and low-fat meat products, and a lower intake of sugar-rich foods. Motivation: Counseling Assessment: Questionnaires, questionnaire-based dietary index	Delivery: In a group or individualized, supervised exercise program. Components: PA of moderate-intensity, at least for 150 min weekly. Motivation: Free access to public swimming pools. Assessment: Self-reporting	Miscarriages or termination of pregnancy (7/6)
Bruno, 2016 [62]	Dietitian	From GW 9–12 until GW 36	Delivery: At enrollment, a counseling session lasting approximately 1 h with a dietitian prescribing a personalized dietary intervention. Four additional follow-up sessions. Components: Decreased intake of foods with a high GI and a high saturated fat content and replacement of them with healthier nutrition based on taste and preferences. A diet based on the high consumption of plant foods, cereals, legumes and fish, with olive oil as the main source of fat, and moderate to no consumption of red wine. A low-glycaemic, low-saturated fat diet with a total intake of 1500 kcal/day divided into three main meals, and three snacks; additionally, daily intake of 300 kcal, and 200 kcal for overweight and obese participants, respectively, due to PA program. Targets for daily dietary macronutrients composition: 55% carbohydrates at least 225 g (ketosis prevention) consisting of 80% complex carbohydrates with low GI, and 20% simple carbohydrates with high GI; 20% protein consisting of 50% animal, and 50% vegetable; 25% fat, consisting of 12% mono-unsaturated, 7% polyunsaturated, 6% saturated; and low intake of saturated fat. Motivation: Telephone calls, counseling Assessment: Questionnaires	Delivery: Group exercise program, at least three times a week. Components: PA of moderate intensity for 30 min each time, according to the ACOG 2002 and the ACSM guidelines. Motivation: Telephone calls, counseling Assessment: Pedometer, talk test	Miscarriages (7/6)
Kennelly, 2018 [17]	Researchers	From GW 12–17 until GW 34	Delivery: Face-to-face education sessions individually or in pairs, smartphone application, emails every two weeks with SMART goals. Components: Healthy recommended approximately eucaloric diet in pregnancy, replacing high-GI with low-GI foods. Motivation: Food diaries quantifying GI and GL, smartphone application, encouragement. Assessment: Self-reported, food diaries, and nutrition calculation software.	Delivery: Exercise program between five and seven days per week. Components: Exercise of moderate intensity for 30 min divided into three or two periods of 10 or 15 min, respectively, according to the ACOG guidelines. Motivation: Smartphone application Assessment: Self-reported, pregnancy exercise and lifestyle surveys	Miscarriages (1/0), congenital anomaly (3/1), pretern delivery and neonatal death (1/0), maternal parvovirus infection (0/1), hyperemesis gravidarum (0/1)
Chan, 2018 [13]	Dietitian, exercise instructor	From GW ≤ 12 until GW 24	Delivery: Bi-weekly face-to-face or phone consultations in the first two months, and monthly face-to-face consultations afterwards till the end of the intervention. Components: An individualized menu plan aimed at achieving a varied balanced diet with an emphasis on fruit and vegetables consumption, and intake of moderate-carbohydrate, low-fat, low-GI and low-caloric products in appropriate portions. Also, advice on the use of dietary supplements and managing pregnancy discomforts. Motivation: Telephone calls, advice, booklet, emails Assessment: Food records, questionnaires, nutrition calculation software, diet adherence score.	Delivery: A supervised exercise program at least three times a week. Components: Based on international guidelines; a session of 30 min of easy to moderate intensity of low-impact aerobics PA. Motivation: Support Assessment: Questionnaires, number of accomplished exercise sessions, PA adherence score	Miscarriages (13/13), pretern delivery (1/1)
Ferrara, 2020 [66]	Dietitians	From 14.3 GW (13.3–15.1) until GW 27.3	Delivery: Core intervention of 13 weekly individual sessions. First and last sessions in person, and 11 remaining sessions by telephone. Components: Eating healthy foods in appropriate portion sizes, total caloric intake, and calories from fat. Motivation: Motivational interviewing techniques, a step-wise, phased approach to behaviour change. Assessment: Workbook, personalised graphs, 24 h dietary recalls	Delivery: Exercise program weekly. Components:150 min per week of moderate-intensity to vigorous intensity PA Motivation: Motivational interviewing techniques, a step-wise, phased approach to behaviour change. Assessment: Accelerometer	Pregnancy loss (5/4)
Lin, 2020 [18]	Researchers	From GW < 8 until GW 24–28	Delivery: One face-to-face education session with an interventionist at the onset of intervention and continuous educational messages delivered via a WeChat public account at a frequency of twice per week. Components: Encouragement to consume vegetables, fruits, high-fiber wholegrain products, low-fat dairy products, and avoid foods rich in sugar and saturated fatty acids, among other guidance. Motivation: Encouragement, messages Assessment: N/a	Delivery: Exercise program at a frequency of three to four times per week. Components: Approximately 30 min of moderate-intensity PA Motivation: Encouragement, messages Assessment: N/a	Early pregnancy loss (5/4)
Li, 2021 [64]	Researchers	ND	Delivery: Individualized medical nutrition guidance Components: Instruction in choosing appropriate foods, and preparation methods for each meal (including vegetables, fruits, and meat). Recommendation to eat less at each meal, and more often at a frequency of six daily meals (three main meals and three additional meals). Energy distribution per each meal category: 15% to 20% for breakfast, 20% to 30% for lunch, 20% to 30% for dinner, and 15% to 30% for extra meals. Calculation of total energy intake of each participant according to pre-pregnancy BMI; increase in the average daily energy intake by 200 kcal on this basis during the second and third trimester of pregnancy, including 50% to 55% carbohydrate, 25% to 30% fat, and 15% to 20% protein. Motivation: Choice of foods Assessment: Food record, anthropometry	Delivery: Individualized exercise program Components: Walking for 15–20 min after meals. Continued engaged pre-pregnancy aerobic activities (i.e., swimming, yoga), according to individual’s preferences and physical condition for 30–40 min. Motivation: Continuation of preferable activities Assessment: Anthropometry	ND
Liu, 2021 [63]	Dietitian	From GW 8–12 until delivery	Delivery: Individual sessions of face-to face instruction and video teaching for 1 h every 2–4 weeks from the first intervention until the end of pregnancy. Components: A hypocaloric, low-glycaemic, low-saturated fat diet with a sample meal plan and recipes. Individualized diet according to pre-pregnancy BMI and PA. For overweight with light PA, recommended 25–30 kcal/kg of ideal body weight (IBW); for obese women with light PA, recommended 20–25 kcal/kg of ideal body weight. Distribution of the daily energy intake as carbohydrate 50–55%, protein 15–20%, and fat 25–30%. Suggested energy for the first trimester not below 1500 kcal/day, and for the second trimester not less than 1600 kcal/day. Motivation: Follow-up through WeChat for participants unavailable for meetings with the dietitian. Assessment: Dietary log	Delivery: Individualized exercise guidance, at least five days each week. Components: Moderate-intensity PA with brisk walking, swimming, dancing, pregnancy yoga, or other aerobic exercises for at least 30 min a day. Motivation: WeChat Assessment: Exercise log, HR monitoring by talking pulse	Miscarriages (19/32)
Ding, 2021 [7]	Dietitian, clinical nutritionist	From GW < 12 until GW 24–28	Delivery: Personalized dietary and exercise guidelines based on the guide for diagnosis and treatment of GDM2014, social media platform, personalized nutrition care Components: IBW energy requirement calculator, and consideration of pregnancy stage. For BMI 24–27.9 and early pregnancy, EER 25–30 kcal/kg; for BMI 24–27.9 and mid-pregnancy, EER 200 kcal in addition to previous intake; for BMI ≥ 28 and early pregnancy EER 20–25 kcal/kg; and for BMI ≥ 28 and mid-pregnancy, EER 200 kcal in addition to previous intake. Minimum energy intake not less than 1500 kcal at early pregnancy, and 1800 kcal at mid-pregnancy. Daily macronutrient components of healthy recommended diet: 50–60% carbohydrate, <30% fat, and 1.0–1.3 g/kg protein. Motivation: An available dietitian, counseling, social media platform Assessment: Dietary surveys, questionnaires	Delivery: Daily exercise program Components: Walk for at least 6000 steps per day Motivation: An available dietitian, counseling, social media platform Assessment: Smartphone data, questionnaires	Early spontaneous abortion (6/5)
Deng, 2022 [8]	Nutritionist, exercise experts, nurses	From GW 14 until GW 24–28	Delivery: One-on-one explanation with an average of 20 min per person, educational manuals Components: Per capita calculation of daily intake of various foods, daily energy intake, daily intake of the three major nutrients, and energy ratio. Energy requirements individually calculated, with 50% to 60% of E% coming from carbohydrates, 20 to 30 E% from fats and 15 to 20 E% from protein. Encouragement to select high-fiber bread, wholemeal products or multigrain rice as staple food; to consume more vegetables and fewer high-sugar fruits; to select fat-free or low-fat versions of milk and milk products; and to use moderate amounts of oil in cooking and baking. Presentation of sample meals and food model samples provided to the women at enrollment. Advice on choosing the favorite food of the same kind through food exchange. Motivation: Advice, social media platform Assessment: Diaries, questionnaires	Delivery: Exercise program at least five days a week. Components: According to the recommendations of ACOG (2015); 20–30 min of moderate-intensity exercise including walking, yoga, and gestational gymnastics designed by exercise experts; in situ steps, upper limb and shoulder back training, and hip 3D exercises. Motivation: Advice, social media platform Assessment: Diaries, questionnaires	None reported
Sadiya, 2022 [10]	Dietitians	From GW ≤ 12; 12 GW	Delivery: Face-to-face individualized dietary consultations. Components: Increased consumption of whole grains, vegetables, fruits, and lower intake of processed food, and simple carboxylates, according to ADA recommendations. Target macronutrient composition: 50–55% carbohydrates, 25–30% fat, and 20% protein. Motivation: Telephone calls, motivational interviewing, SMART goals Assessment: Food recalls, questionnaires, food log	Delivery: A choice of a weekly or daily exercise program. Components: Weekly moderate-intensity PA of 150 min or a daily minimum of 10,000 steps; 1 h and 40min daily activity. Motivation: Telephone calls, motivational interviewing, SMART goals Assessment: Self-monitoring, smartphone pedometer application	Miscarriages (2/2)

GDM, gestational diabetes mellitus; GW, gestational week; IOM, Institute of Medicine; ACOG, American College of Obstetricians and Gynecologists; ACSM, American College of Sports Medicine; ADA, American Diabetes Association; BMI, body mass index; GWG, gestational weight gain; IBW, ideal body weight; SMART, specific, measurable, action-oriented, realistic, timed; EER, estimated energy requirement; E, total energy; FCM, food choice map; kcal, kilocalories; kg, kilogram; g, gram; GI, glycaemic index; GL, glycaemic load; PA, physical activity; MET, metabolic equivalent task; HR, heart ratio; min, minute; DVD, digital video disk; 3D, three-dimensional; ND, no data; N/a, not applicable; h, hour.

**Table 10:** Quality of reporting for eligible studies with dietary interventions.

First Author, Publication Year	Random Sequence Generation (Selection Bias)	Allocation Concealment (Selection Bias)	Blinding of Participants and Personnel (Performance Bias)	Blinding of Outcome Assessment (Detection Bias)	Incomplete Outcome Data (Attrition Bias)	Selective Reporting (Reporting Bias)	Other Bias
Korpi-Hyövälti, 2011 [40]	L	L	?	L	L	L	L
Quinlivan, 2011 [43]	L	L	?	L	L	L	L
Walsh, 2012 [41]	L	L	?	L	L	L	L
McCarthy, 2016 [42]	L	L	?	L	L	L	L
Yi Zhang, 2019 [45]	L	L	H	L	L	L	L
Al Wattar, 2019 [20]	L	L	H	L	L	L	L
Okesene-Gafa, 2019 [44]	L	L	H	L	L	L	L
Melero, 2020 [3]	L	?	?	L	L	L	L
Basu, 2021 [4]	L	L	H	L	L	L	L
Dong-Yao Zhang, 2022 [16]	L	L	?	L	L	L	L

H, high risk; L, low risk; ?, unclear.

**Table 11:** Quality of reporting for eligible studies with exercise intervention.

First Author, Publication Year	Random Sequence Generation (Selection Bias)	Allocation Concealment (Selection Bias)	Blinding of Participants and Personnel (Performance Bias)	Blinding of Outcome Assessment (Detection Bias)	Incomplete Outcome Data (Attrition Bias)	Selective Reporting (Reporting Bias)	Other Bias
Do Nascimento, 2011 [52]	L	L	?	L	L	L	L
Oostdam, 2012 [51]	L	L	?	L	L	L	L
Price, 2012 [54]	L	L	?	?	H	L	L
Barakat, 2013 [46]	L	L	H	L	L	L	L
Ruiz, 2013 [49]	L	L	?	L	L	L	L
Nobles, 2015 [55]	L	L	?	L	L	L	L
Bisson, 2015 [53]	L	L	?	L	L	L	L
Seneviratne, 2015 [57]	L	L	H	L	L	L	L
Perales, 2016 [48]	L	L	H	L	H	L	L
Krohn Garnæs, 2016 [50]	L	L	?	L	L	L	L
Guelfi, 2016 [56]	L	L	?	?	L	L	L
Wang, 2017 [58]	L	L	H	L	L	L	L
Daly, 2017 [47]	L	L	H	L	L	L	L

H, high risk; L, low risk; ?, unclear.

**Table 12:** Quality of reporting for eligible studies with diet-plus-exercise interventions.

First Author, Publication Year	Random Sequence Generation (Selection Bias)	Allocation Concealment (Selection Bias)	Blinding of Participants and Personnel (Performance Bias)	Blinding of Outcome Assessment (Detection Bias)	Incomplete Outcome Data (Attrition Bias)	Selective Reporting (Reporting Bias)	Other Bias
Luoto, 2011 [5]	L	L	H	L	L	L	L
Vinter, 2011 [59]	L	L	?	L	L	L	L
Harrison, 2013 [11]	L	L	?	L	L	L	L
Petrella, 2013 [61]	L	L	?	L	L	L	L
Dodd, 2014 [65]	L	L	?	L	L	L	L
Hui, 2014 [67]	L	L	H	L	L	L	L
Poston, 2015 [60]	L	L	H	L	L	L	L
Koivusalo, 2015 [6]	L	L	?	L	L	L	L
Bruno, 2016 [62]	L	L	?	L	H	L	L
Kennelly, 2018 [17]	L	L	H	L	L	L	L
Chan, 2018 [13]	L	L	H	L	L	L	L
Ferrara, 2020 [66]	L	L	?	L	L	L	L
Lin, 2020 [18]	L	L	?	L	L	L	L
Li, 2021 [64]	L	L	?	L	L	?	L
Liu, 2021 [63]	L	L	H	L	L	L	L
Ding, 2021 [7]	L	L	H	L	L	L	L
Deng, 2022 [8]	L	L	H	L	L	L	L
Sadiya, 2022 [10]	L	L	H	L	L	L	L

H, high risk; L, low risk; ?, unclear.

## Data Availability

Data are contained within the article or Supplementary Material.

## Supplementary Materials

The following supporting information can be downloaded at: https://www.mdpi.com/article/10.3390/jcm12227038/s1. Table S1: Search Strategy; Table S2: Assessment of the exercise intervention based on the CERT tool; Table S3: Diagnostic modalities of GDM in eligible trials with dietary intervention; Table S4: Diagnostic modalities of GDM in eligible trials with exercise intervention; Table S5: Diagnostic modalities of GDM in eligible trials with dietary plus exercise intervention; Table S6: Subgroup and sensitivity analyses for dietary intervention; Table S7: Meta-regression for GDM OR in dietary intervention; Table S8: Subgroup and sensitivity analyses for exercise intervention; Table S9: Meta-regression for GDM OR in exercise intervention; Table S10: Subgroup and sensitivity analyses for diet-plus-exercise intervention; Table S11: Meta-regression for GDM OR in diet-plus-exercise intervention; Table S12: GRADE evaluation of overall evidence for dietary intervention; Table S13: GRADE evaluation of overall evidence for exercise intervention; Table S14: GRADE evaluation of overall evidence for diet-plus-exercise intervention.
