# Supplementary material for: Effectiveness of Lifestyle Interventions during Pregnancy on Preventing Gestational Diabetes Mellitus in High-Risk Women: A Systematic Review and Meta-Analyses of Published RCTs
Source: J Clin Med. 2023 Nov 10;12(22):7038. doi: 10.3390/jcm12227038 (PMC10672732; doi:10.3390/jcm12227038)
Supplement: Supplementary file 1 [file jcm-12-07038-s001.zip › jcm-2677856-supplementary.pdf]

Effectiveness of lifestyle interventions during pregnancy on preventing gestational diabetes mellitus in high-risk women: a systematic review and meta-analyses of published RCTs

Georgios I. Tsironikos[1], Petros Potamianos[2], George E. Zakynthinos[3], Vasiliki Tsolaki[4*], Athina Tatsioni[5], Alexandra Bargiota[6]

[1] Department of Medicine, University of Ioannina, University Campus, 45110 Ioannina, Greece, E-mail: gtsironikos@gmail.com
[2] Department of Gastroenterology, University Hospital of Larissa, Faculty of Medicine, University of Thessaly, Mezourlo, 41335 Larissa, Greece, E-mail: petpot92@gmail.com
[3] 3rd Department of Cardiology, "Sotiria" Chest Diseases Hospital, Medical School, National and Kapodistrian University of Athens, 11527 Athens, Greece, E-mail: gzakynthinos2@gmail.com
[4] Department of Critical Care, University Hospital of Larissa, Faculty of Medicine, University of Thessaly, Mezourlo, 41335 Larissa, Greece, E-mail: vasotsolaki@yahoo.com
[5] Department of Research Unit for General Medicine and Primary Health Care, University of Ioannina, University Campus, 45110 Ioannina, Greece, E-mail: atatsion@uoi.gr
[6] Department of Internal Medicine-Endocrinology, University Hospital of Larissa, Faculty of Medicine, University of Thessaly, Mezourlo, 41335 Larissa, Greece, E-mail: abargio@med.uth.gr

*Corresponding Author
E-mail: vasotsolaki@yahoo.com

Supplementary material
Table S1. Search Strategy

PubMed	((diet OR nutrition) OR (exercise OR "physical activit*" OR workout)) AND (diabetes AND (gestational OR pregnancy)) AND ("Clinical Trials as Topic"[Mesh] OR "randomized controlled trial"[pt] OR "controlled clinical trial"[pt] OR randomized[tiab] OR placebo[tiab] OR randomly[tiab] OR trial[tiab])<br>with PubMed publication date Between Jan 1950 and Aug 2022
CENTRAL	((diet OR nutrition) OR (exercise OR "physical NEXT activit*" OR workout)) AND (diabetes AND (gestational OR pregnancy)) with Cochrane Library publication date Between Jan 1950 and Aug 2022 (Word variations have been searched)
Scopus	TITLE-ABS-KEY ((diet OR nutrition) OR (exercise OR "physical activit*" OR workout) AND (diabetes AND (gestational OR pregnancy))) AND (LIMIT-TO (DOCTYPE , "ar")) AND (LIMIT-TO (EXACTKEYWORD , "Female") OR LIMIT-TO (EXACTKEYWORD , "Pregnancy")) AND (LIMIT-TO (LANGUAGE , "English")) AND (EXCLUDE (PUBYEAR , 2023))

Table S2. Assessment of the exercise intervention based on CERT tool

First author, Publication year, Country	1) Equipment	2) Provider	3) Performance	4) Supervision	5) Adherence	6) Motivation	7) Progressing	8) Description, reproduction	9) Home component	10) Nonexercise components	11) Adverse events	12) Location	13) Dosage	14) Tailoring to individual	15) Starting level	16) How well
Do Nascimento, 2011, Brazil	ND	Physiotherapist	In a group or individually	Supervised	Exercise journal	Counselling	ND	ND	Five times weekly	Counselling on GWG, nutrition	None reported	Prenatal Outpatient Clinic of the Women's Integral Healthcare Centre	ACOG (2002); strengthen program of 40 min, light- to moderate-intensity, weekly	Generic	ND	62.5% adherence and 60% 9 – 16 weeks home PA
Oostdam, 2012,	Cycle erg	Physiother	Individually	Supervised	Accelerometer,	Counselling	Number of	ND	ND	ND	None reported	Department of Physiothera	Light intensity warming-	Training intensity carefully	Maximal muscle strength	Close supervision of a

Netherlands	ometers, treadmills, cross-trainers, stationary rowing machines, free weights, accelerometer	apist			MET from the ACSM		repetitions, talk test, Borg Scale until 12					py in the participating hospitals	up for 5–10 min., such as slow cycling at a level of 50 Watt. 40 min of 1 or 2 aerobic exercises and 4 to 6 strength exercises. Cool-down for 5–10 min by slowly reducing the activity.	and individually controlled.	and aerobic capacity, Valsalva maneuver during resistance exercise, one Repetition Maximum (1RM; the heaviest weight that can be lifted once) predicted from the Oddvar Holten diagram.	physiotherapist under the guide of ACOG

Price, 2012, USA	Treadmills, elliptical trainers, stationary bicycles, weight machines, exercise balls	Researchers	Both in a group and individually	Supervised	Borg Scale, temperature and humidity rates, sit-and-reach test, questionnaire	ND	A program at moderate intensity (12 – 14 on Borg Scale)	ND	ND	Dietary advice	Intervention Group: anxiety with exercise (n = 1), preterm pregnancy (n = 1), pain from leiomyomas (n = 1)	ND	ACOG; aerobic training of 45 – 60 min, 4 times per week, at moderate intensity. Step aerobics on the 1st day, walked as a group on the 2nd day, and circuit training on a 3rd day, of 1–10 min of aerobic exercise, alternating with an equal time interval of weight	Generic	Aerobic fitness test, submaximal strength assessment by lifting a 7-kg medicine ball from floor to waist high as many times possible within 1 min	Author's log of exercise activities and attendance

													training, using a weight that allowed one set of 20 repetitions. End with 5 min of hamstring, quadriceps, and calf stretching. Also, a brisk walk individually, 30- to 60-min, once weekly.			
Barakat, 2013, Spain	Bar-bells, the rab and	Fitness specialist,	In a group	Supervised	HR monitor, Borg Scale	Music, and airy, well-lighted exercise room	Moderate exercise intensity	ND	ND	ND	Under medical follow-up	Exercise room at the Hospital	3 days/week, 50–55 min/ session. Warm-up and cool-	One size fit all	ND	Training sessions in a group of 10–12 women, carefull

	s, HR monitor	obstetrician				at the Hospital							down period, both of 10–12 min; walking and light, static stretching of most muscle group. Main part of 25–30 min; moderate-intensity resistance exercises at full range of major muscle groups motion. One set of pelvic tilting in standing position			y supervised by qualified fitness specialist with the assistance of an obstetrician.

													and exercises using bar bells or low-to-medium resistance elastic bands (Therabands). In addition, one session/week of aerobic choreography dance in sections of 3–4 min with 1 min breaks of stretching and relaxation activities.			

Ruiz, 2013, Spain	Bar-bells (3 kg), bench, elastic bands (therabands), HR monitor	ND	In a group	Supervised	HR monitors, and Borg conventional	ND	Light to moderate exercise intensity	ND	ND	ND	Intervention Group: threat of prematurity (n=14), persistent bleeding (n=7); Control group: threat of prematurity (n=11), persistent bleeding (n=9)	Hospital Universitario de Fuenlabrada, Madrid, Spain	Light to moderate-intensity aerobic and resistance exercise, 3 times a week, 50-55 min per session. Start and finish with 10-min warm-up and 10-min cool-down, respectively, with same exercises of light intensity walking and static stretching of most	HR consistently less than 60% of age predicted maximum HR (208 - [0.7 * age in years]), and rate of RPE n range from 10 to 12 (fairly light to somewhat hard, respectively).	ND	Total pleasure with the intervention, adherence> 97%, intention to be physically active during future pregnancies.

													muscle groups, plus relaxation and pelvic floor exercises at cool-down period. Core section of 25 to 30 min of moderate intensity aerobic exercises once a week; low-impact aerobic dance activities for 3 to 4 min with 1-min breaks, with			

													stretching and relaxation of upper and lower limbs. Resistance exercises twice a week; performance through the full range of motion, engaging major muscle groups, strengthening the muscles used in labor and the pelvic floor (3^{rd}trimester), and			

													supine position on the floor exercises up to 2 min.			
Nobles, 2015. USA	Digital pedometer	Health educators	ND	ND	Questionnaires, manuals	Booster telephone calls, mailed tip sheets	Safe progress toward the overall PA goal; weekly increasement by 10% of spent time at moderate	ND	ND	Counseling of IOM guidelines for appropriate nutrition and GWG.	Medical records	ambulatory obstetric practices of Baystate Medical Center, western Massachusetts	≥ 30 min of moderate-intensity PA on most days of the week. Specific self-selected activities including dancing, walking, and yard work.	Generic	Tailoring questionnaire	62.1% of participants meting the College's guidelines for PA, adherence as returning completed questionnaires, 95% of participants satisfied with

							intensity PA									information received
Bisson, 2015, Canada	Stationary ergocycle, treadmill, exercising balls, strength equipment, HR monitors, acceler	Kinesiologists	Individually	Supervised	Self-monitoring with HR monitors, Borg Scale, exercise log, accelerometry, questionnaire, MET	Counseling, modification of PA, pamphlet	Targets at 70% of peak HR (fitness test), progressively increasement PA from 15 min of 1st week to 30 min by the end of the	ND	ND	ND	None reported	Heart Disease prevention Pavilion (University Institute of Cardiology and Pneumology of Quebec)	ACSM guidelines; moderate-intensity exercise sessions of 1h, 3 times weekly. Warm-up 5–10 min on a stationary ergocycle. 15–30 min treadmill walk. 20 min muscular work-out with dynamic exercises for both lower and upper	Intensity adjusted to participants' tolerance level.	Cardiorespiratory testing in obese pregnant women. Assessment for PA, anthropometry, fitness and fetal growth. Start with 1 set of 10–15 repetitions per exercise and progressed to 2 sets of 15 repetitions.	Documentation of PA at GW 36. Participants' percentages and accomplished level of prescribed sessions: 60% ≥ 50%, and 20% ≥ 75%.

	ometer						1st month.						limbs using the participant's own body weight, small weights, exercising balls and strength equipment with selective charge. Cool-down period.			
Seneviratne, 2015, New Zealand	Magnetic stationary bicycles, HR mo	Exercise physiologist	Individually	Unsupervised	HR monitors	An available exercise physiologist for help with exercise-	HR in exercise sessions at moderate intensity	Written program of prescription	Home-based cycling program	ND	None reported	At home	5-min warm-up and cool-down period at low intensity. Frequency varying between three and five	According to stage of pregnancy	ND	Download of HR monitor data

	nitors					related problems.							sessions per week, and duration of moderate-intensity exercise between 15 and 30 min per session, according to stage of pregnancy.			
Perales, 2016, Spain	HR monitors, light weights, fit ball, dumb	Fitness instructors	In a group	Supervised	HR monitors	ND	Narrow HR target at high end of moderate–intensity; 55%–	Visualization techniques	ND	ND	Intervention Group: risk of prematurity(n=4), obstetric complications (n=3); Control Group: risk of prematurity (n=5), obstetric	Hospital Universitario de Fuenlabrada, Madrid, Spain	Three aerobic and strength training sessions per week, of 55–60 min per session. Constant duration and structure.	Generic	ND	HR values every 2, 15 min after of sitting while taking deep breaths.

	bells						60% of HRR				complications (n=4)		Start with a 5- to 7-min warm-up consisting of walking at different intensities, mild static stretching of most muscle groups, joint mobility exercises, and locomotion games. Aerobic activities for 25–30 min of different exercises and music choreogra			

													phies. Next, the strength part of the session, engaging major muscles groups with the aim of improvin g general muscle strength and preventin g common muscle imbalance s during pregnanc y (low back pain, sciatica, and kyphosis), and improvin			

													g balance, pelvis mobility, and body awareness, as well as pelvic floor muscle exercises for prevention of urinary incontinence. End with a 5- to 10-min cool-down period including relaxing exercises, static stretching, and partner massages.			

Krohn Garnæs, 2016, Norway	Treadmill	Physiotherapist	In a group	Supervised	Training diary, Borg Scale	Motivational interview session, and encouragement	Endurance exercise at 80% of the maximum capacity, according to the Borg Scale 12-15.	ND	50 min at least one weekly (35 min of endurance training and 15 min of strength exercises), and daily pelvic floor muscle exercises	ND	None reported	St. Olvas Hospital	Three times a week, 35 min of moderate-intensity endurance exercise and 25 min of strength training.	Adjustment to each woman's strength level.	ND	Late pregnancy: physically active ≥ 30 min/day 61%, and regular exercise training 77%
Guelfi, 2016, Australia	Upright cycle ergometer, acc	Exercise physiologist	Individually	Supervised	Accelerometer, questionnaire	ND	Progressively increased session's duration by 5-	ND	Home-based stationary cycling program	ND	Intervention Group: pregnancy loss (n=1); Control Group: pregnancy loss (n=2)	At home	Sessions three times each week. Start with a 5-min warm up consisting of	Tailored to the individual	ND	Supervised sessions by an exercise physiologist monitoring the duration

	elerometer						min every 2–3 weeks, from 20 to 30 min to a max of 60 min. Degree of progression according to the baseline fitness level and ongoing preg						pedaling at an intensity equated to 55–65% of age-predicted maximum HR and a rating of 9–11 on the Borg Scale. Subsequent period divided into 5-min periods of continuous moderate-intensity cycling, alternating with 5-min periods of interval cycling; two types			and intensity of exercise.

							nancy symptoms.						of intervals: an increase in pedaling rate for 15 seconds (sec), and an increase in cycling resistance for 30 sec repeated every 2 min. End with 5-min cool-down by light stretching.			
Wang, 2017, China	Stationary bike	Researchers	ND	Supervised	Questionnaires, Borg Scale assessing	ND	Progressive increase of the exerc	ND	ND	ND	Standard prenatal care for all women. Recording and review of	Peking University First Hospital	Exercise at the beginning of the intervention at the lower	Tailored to the individual	Measurements of each participant's height to the nearest	Questionnaire, metabolic equivalents of task

					the RPE score, records based on the RPE score		ise duration to 45-60 min, adding 5 min to the intervals or the continuous phases of cycling, based on individual abilities				cervical length at each examination and exclusion of women with a cervical length <25 mm.		calculated limit, based on the maximum predicted HR for age, progressively increased with the progress of the program, at least 3 days a week.		0.5 cm without shoes, weight accurate to 0.1 kg with light clothing, and BMI calculation as kg/m².	min/week
Daly, 2017, Ireland	Weights	Resear	In a group	Supervised	HR monitorin	Secret Facebook	According to the	ND	ND	Facebook group	None reported	Coombe Women and Infants	50–60 min of exercise sessions;	Exercises scaled	ParMed-X for pregnanc	Attendance recorde

		chers			g, Borg Scale	group goal-setting, class in a group, journaling, taught weekly, on a choice of days, and h, varied each day, and a "kids' corner" with toys and a mat and playpen.	ACOG			lifestyle advice, pamphlet with information on healthy eating during pregnancy based on national guidelines		University Hospital, Dublin, Ireland	10-min warm-up focused on core and pelvic floor exercises; 15–20 min of resistance or weights working important muscle groups for a healthy pregnancy and birth recovery (lower limb, upper limb, back, and core muscle groups); 15–20 min	according to the ability, SMART personal goals	y form to rule out contraindications to exercise in pregnancy	d by both participants and researchers

													of aerobic exercises; and 10-min cool-down.			

ND, no data; GWG, gestational weight gain; ACOG, American College of Obstetricians and Gynecologists; PA, physical activity; MET, metabolic equivalent of task; ACSM; American College of Sports Medicine; Min, minute; 1RM; incomplete rest method; kg, kilogram; HR, heart ration; RPE; rate of perceived exertion; IOM, institute of medicine; h, hour; GW, gestational week; HRR, heart rate recovery; St, Saint; BMI, body mass index; SMART, specific measurable achievable relevant and time-bound

Table S3. Diagnostic modalities of GDM in eligible trials with dietary intervention

First author, publication year	Diagnostic test for GDM	Gestational weeks of diagnostic test performance
Korpi-Hyövälti, 2011	2-h 75-gr OGTT	8-12
Quinlivan, 2011	2-h 75-gr OGTT	24-28
Walsh, 2012	1-h 50-gr OGTT; FBG 100-gr OGTT, 1-h 100-gr OGTT, 2-h 100-gr OGTT, 3-h 100-gr OGTT	28
McCarthy, 2016	1-h 50-gr OGTT; FBG 75-gr OGTT, 1-h 75-gr OGTT, 2-h 75-gr OGTT	26-28
Yi Zhang,2019	2-h 75-gr OGTT	24-28
Al Wattar, 2019	FBG 75-gr OGTT, 2-h 75-gr OGTT	24-28
Okesene-Gafa, 2019	FBG 75-g OGTT, 1-h 75-gr OGTT, 2-h 75-gr OGTT	26-28
Melero, 2020	FBG 75-gr OGTT, 1-h 75-gr OGTT, 2-h 75-gr OGTT	24-28
Basu, 2021	1-h 50-gr OGTT; FBG 100-gr OGTT, 1-h 100-gr OGTT, 2-h 100-gr OGTT, 3-h 100-gr OGTT	24-28
Dong-Yao Zhang, 2022	FBG 75-gr OGTT, 1-h 75-gr OGTT, 2-h 75-gr OGTT	25-28

GDM, gestational diabetes mellitus; h, hour; gr, grams; OGTT, oral glucose tolerance test; FBG, fasting blood glucose

Table S4. Diagnostic modalities of GDM in eligible trials with exercise intervention

First author, publication year	Diagnostic test for GDM	Gestational weeks of diagnostic test performance
Do Nascimento, 2011	ND	ND
Oostdam, 2012	FBG; HbA1c	24, 32
Price, 2012	FBG 100-gr OGTT, 1-h 100-gr OGTT, 2-h 100-gr OGTT, 3-h 100-gr OGTT	ND
Barakat, 2013	FBG 75-gr OGTT, 1-h 75-gr OGTT, 2-h 75-gr OGTT	24-26
Ruiz, 2013	ND	ND
Nobles, 2015	1-h 50-gr OGTT; FBG 100-gr OGTT, 1-h 100-gr OGTT, 2-h 100-gr OGTT, 3-h 100-gr OGTT	24-28
Bisson, 2015	ND	28
Seneviratne, 2015	FBG 75-gr OGTT, 2-h 75-gr OGTT	ND
Perales, 2016	1-h 50-gr OGTT; FBG 100-gr OGTT, 1-h 100-gr OGTT, 2-h 100-gr OGTT, 3-h 100-gr OGTT	24-28
Krohn Garnæs, 2016	FBG 75-gr OGTT, 2-h 75-gr OGTT	24-28
Guelfi, 2016	FBG 75-gr OGTT, 2-h 75-gr OGTT	26-28
Wang, 2017	FBG 75-gr OGTT, 1-h 75-gr OGTT, 2-h 75-gr OGTT	24-28
Daly, 2017	FBG 75-gr OGTT, 1-h 75-gr OGTT, 2-h 75-gr OGTT	24-28

GDM, gestational diabetes mellitus; ND, no data; FBG, fasting blood glucose; HbA1c, hemoglobin A1c; h, hour; gr, grams; OGTT, oral glucose tolerance test

Table S5. Diagnostic modalities of GDM in eligible trials with dietary plus exercise intervention

First author, publication year	Diagnostic test for GDM	Gestational weeks of diagnostic test performance
Luoto, 2011	FBG 75-gr OGTT, 1-h 75-gr OGTT, 2-h 75-gr OGTT	26-28
Vinter, 2011	2-h 75-gr OGTT	12-14, 28-30, 34-36
Harrison, 2013	FBG 75-gr OGTT, 1-h 75-gr OGTT, 2-h 75-gr OGTT	12-15, 26-28
Petrella, 2013	FBG 75-gr OGTT, 1-h 75-gr OGTT, 2-h 75-gr OGTT	16-18, 24-28
Dodd, 2014	FBG 75-gr OGTT, 2-h 75-gr OGTT	12-14, 24-28
Hui, 2014	FBG 75-gr OGTT, 1-h 75-gr OGTT, 2-h 75-gr OGTT	24-28
Poston, 2015	FBG 75-gr OGTT, 1-h 75-gr OGTT, 2-h 75-gr OGTT	27^{+6d}-28^{+6d}
Koivusalo, 2015	FBG 75-gr OGTT, 1-h 75-gr OGTT, 2-h 75-gr OGTT	24-28
Bruno, 2016	FBG 75-gr OGTT, 1-h 75-gr OGTT, 2-h 75-gr OGTT	16-18, 24-28
Kennelly, 2018	FBG 75-gr OGTT, 1-h 75-gr OGTT, 2-h 75-gr OGTT	28
Chan, 2018	FBG 75-gr OGTT, 1-h 75-gr OGTT, 2-h 75-gr OGTT	24-28
Ferrara, 2020	1-h 50-gr OGTT; FBG 100-gr OGTT, 1-h 100-gr OGTT, 2-h 100-gr OGTT, 3-h 100-gr OGTT	8-15, 29-38
Lin, 2020	FBG 75-gr OGTT, 1-h 75-gr OGTT, 2-h 75-gr OGTT	24-28
Li, 2021	1-h 50-gr OGTT; FBG 100-gr OGTT, 1-h 100-gr OGTT, 2-h 100-gr OGTT, 3-h 100-gr OGTT	24-28
Liu, 2021	FBG 75-gr OGTT, 1-h 75-gr OGTT, 2-h 75-gr OGTT	24-28
Ding, 2021	FBG 75-gr OGTT, 1-h 75-gr OGTT, 2-h 75-gr OGTT	24-28
Deng, 2022	FBG 75-gr OGTT, 1-h 75-gr OGTT, 2-h 75-gr OGTT	24-28
Sadiya, 2022	FBG 75-gr OGTT, 1-h 75-gr OGTT, 2-h 75-gr OGTT	24-28

GDM, gestational diabetes mellitus; FBG, fasting blood glucose; gr, grams; OGTT, oral glucose tolerance test; h, hour; d, days

Table S6. Subgroup and sensitivity analyses for dietary intervention

Factor	Subgroup	Number of studies	Odds Ratio (95% CI)	P-value	Cochran's Q statistic	I^2 (95%CI)	Test of difference P-value
**Subgroup analyses**							
Similar Countries	Oceania	3	0.75 (0.29, 2.00)	0.57	10.54	81% (48, 93%)	0.76
	Great Britain	2	0.65 (0.49, 0.87)	0.003	0.04	0% (n/a)	
	China	2	0.63 (0.18, 2.20)	0.46	3.87	74% (n/a)	
	Finland	1	3.25 (0.32, 33.41)	0.32	n/a	n/a	
	Spain	1	0.50 (0.27, 0.94)	0.03	n/a	n/a	
	USA	1	0.51 (0.10, 2.61)	0.42	n/a	n/a	
Low education level	More than 10% of participating women	2	0.86 (0.56, 1.33)	0.50	5.95	83% (54, 94%)	0.52
	Up to 10% of participating women	1	0.94 (0.67, 1.32)	0.78	n/a	n/a	
Increased BMI as a risk factor	Included	8	0.78 (0.64, 0.96)	0.02	19.38	64% (0, 95%)	0.24
	Not included	2	0.57 (0.36, 0.93)	0.02	0.43	0% (n/a)	
Intervention duration	More than 20 weeks	3	0.91 (0.59, 1.39)	0.66	7.14	72% (20, 90%)	0.17
	Up to 20 weeks	2	0.54 (0.29, 1.01)	0.05	1.48	33% (n/a)	
Mediterranean diet	Included	2	0.61 (0.46, 0.81)	0.0005	0.48	0% (n/a)	0.06
	Not included	8	0.88 (0.68, 1.15)	0.35	16.71	58% (48, 65%)	

Motivation component in the intervention	Included	9	0.71 (0.58, 0.87)	0.0008	19.40	59% (17, 79%)	0.16
	Not included	1	1.08 (0.62, 1.87)	0.78	n/a	n/a	
**Sensitivity analyses**							
Effect of RCT with the largest sample size	Lower sample size	9	0.73 (0.48, 1.13)	0.16	19.67	59% (19, 79%)	n/a

USA, United States of America; CI, confidence interval; n/a, not applicable

Table S7. Meta-regression for GDM OR in dietary intervention

Covariate	Number of studies	Coefficient b (95% CI)	SE	P-value	t
Baseline risk of GDM	10	-0.5189 (-5.69526, 4.65746)	2.641	0.085	-1.965
Study duration (months)	8	0.019 (-0.00844, 0.04644)	0.014	0.228	1.342

GDM, gestational diabetes mellitus; OR, odds ratio; CI, confidence interval; SE, standard error

Table S8. Subgroup and sensitivity analyses for exercise intervention

Factor	Subgroup	Number of studies	Odds Ratio (95% CI)	P-value	Cochran's Q statistic	I^2 (95%CI)	Test of difference P-value
**Subgroup analyses**							
Similar Countries	North America	3	0.61 (0.33, 1.16)	0.13	0.07	0% (0, 6%)	0.17
	Spain	3	0.59 (0.42, 0.84)	0.003	0.29	0% (0, 6%)	
	Oceania	2	1.10 (0.62, 1.97)	0.74	0.59	0% (n/a)	
	Brazil	1	0.45 (0.17, 1.22)	0.12	n/a	n/a	
	Netherlands	1	0.62 (0.22, 1.76)	0.37	n/a	n/a	
	Norway	1	0.53 (0.16, 1.81)	0.31	n/a	n/a	
	Ireland	1	1.46 (0.62, 3.41)	0.39	n/a	n/a	
	China	1	0.41 (0.24, 0.71)	0.001	n/a	n/a	
Low education level	More than 10% of participating women	8	0.54 (0.42, 0.69)	< 0.00001	1.82	0% (0, 83%)	0.98
	Up to 10% of participating women	1	0.53 (0.16, 1.81)	0.31	n/a	n/a	
Increased BMI as a risk factor	Included	8	0.60 (0.44, 0.82)	0.001	8.39	17% (0, 72%)	0.59
	Not included	5	0.68 (0.50, 0.91)	0.01	2.56	0% (0, 69%)	
Intervention duration	More than 20 weeks	8	0.60 (0.48, 0.75)	< 0.0001	6.41	0% (0, 64%)	0.28
	Up to 20 weeks	5	0.77 (0.52, 1.15)	0.20	3.73	0% (0, 78%)	
Motivation component	Included	8	0.69 (0.51, 0.92)	0.01	5.81	0% (0, 60%)	0.50

in the intervention	Not included	5	0.59 (0.43, 0.81)	0.001	5.05	21% (0, 84%)	
**Sensitivity analyses**							
Effect of RCT with the largest sample size	Lower sample size	12	0.66 (0.52, 0.83)	0.0005	10.75	0% (0, 70%)	n/a
Attrition bias	Low	11	0.64 (0.50, 0.82)	0.0004	11.23	11% (0, 64%)	n/a

CI, confidence interval; n/a, not applicable

Table S9. **Meta-regression for GDM OR in exercise intervention**

**Covariate**	**Number of studies**	**Coefficient b (95% CI)**	**SE**	**P-value**	**t**
Baseline risk of GDM	13	-0.108 (-2.0484, 1.8324)	0.999	0.916	-0.108
Study duration (months)	13	0.001 (- 0.001156, 0.003156)	0.011	0.949	0.066

GDM, gestational diabetes mellitus; OR, odds ratio; CI, confidence interval; SE, standard error

Table S10. **Subgroup and sensitivity analyses for diet plus exercise intervention**

Factor	Subgroup	Number of studies	Odds Ratio (95% CI)	P-value	Cochran's Q statistic	I^2 (95%CI)	Test of difference P-value
**Subgroup analyses**							
Similar Countries	China	6	0.55 (0.33, 0.90)	0.02	15.89	69% (30, 86%)	0.02
	Finland	2	0.90 (0.41, 2.00)	0.79	3.80	74% (n/a)	
	Great Britain	2	0.98 (0.78, 1.23)	0.87	0.31	0% (n/a)	
	Italy	2	0.32 (0.16, 0.60)	0.0005	0.91	0% (n/a)	
	North America	2	0.90 (0.45, 1.80)	0.77	0.77	0% (n/a)	
	Oceania	2	0.92 0.45 1.88	0.82	4.83	79% (0, 84%)	
	Denmark	1	1.16 (0.44, 3.10)	0.76	n/a	n/a	
	UAE	1	0.37 (0.13, 1.03)	0.06	n/a	n/a	
Low education level	More than 10% of participating women	7	0.73 (0.55, 0.95)	0.02	15.80	62% (20, 82%)	0.34
	Up to 10% of participating women	3	0.90 (0.64, 1.26)	0.54	2.53	21% (0, 97%)	
Increased BMI as a risk factor	Included	17	0.89 (0.79, 1.01)	0.06	41.45	61% (36, 81%)	0.003
	Not included	1	0.17 (0.06, 0.51)	0.001	n/a	n/a	
Intervention duration	More than 20 weeks	6	1.09 (0.90, 1.32)	0.84	11.86	58% (2, 82%)	0.03
	Up to 20 weeks	8	0.81 (0.67, 0.97)	0.02	17.85	61% (51, 69%)	
**Sensitivity analyses**							

Effect of RCT with the largest sample size	Lower sample size	17	0.67 (0.52, 0.86)	0.002	40.16	60% (34, 76%)	n/a
Attrition bias	Low	17	0.73 (0.57, 0.93)	0.01	46.33	65% (42, 79%)	n/a

UAE, United Arab Emirates; CI, confidence interval; n/a, not applicable

Table S11. Meta-regression for GDM OR in diet plus exercise intervention

Covariate	Number of studies	Coefficient b (95% CI)	SE	P-value	t
Baseline risk of GDM	18	-1.754 (-3.41412, -0.09388)	0.847	0.055	-2.071
Study duration (months)	17	0.016 (0.00032, 0.03168)	0.008	0.065	1.991

GDM, gestational diabetes mellitus; OR, odds ratio; CI, confidence interval; SE, standard error

Table S12. GRADE evaluation of overall evidence for dietary intervention

Diet for GDM prevention

Patient or population: pregnant women with high risk for GDM
Settings: outpatient
Intervention: Diet

Outcomes	Illustrative comparative risks* (95% CI)		Relative effect (95% CI)	No of Participants (studies)	Quality of the evidence (GRADE)	Comments
	Assumed risk	Corresponding risk				
	Control	GDM				
GDM	**Study population**		**OR 0.73** (0.51 to 1.03)	3109 (10 studies)	⊕⊕⊕⊖ **moderate**[1]	
	**198 per 1000**	**152 per 1000** (112 to 202)				
	**Moderate**					
	**245 per 1000**	**192 per 1000** (142 to 251)				

*The basis for the **assumed risk** (e.g. the median control group risk across studies) is provided in footnotes. The **corresponding risk** (and its 95% confidence interval) is based on the assumed risk in the comparison group and the **relative effect** of the intervention (and its 95% CI).

CI: Confidence interval; **OR:** Odds ratio;

GRADE Working Group grades of evidence
High quality: Further research is very unlikely to change our confidence in the estimate of effect.
Moderate quality: Further research is likely to have an important impact on our confidence in the estimate of effect and may change the estimate.
Low quality: Further research is very likely to have an important impact on our confidence in the estimate of effect and is likely to change the estimate.
Very low quality: We are very uncertain about the estimate.

[1] Significant level of heterogeneity

Table S13. GRADE evaluation of overall evidence for exercise intervention

Exercise for GDM prevention

Patient or population: pregnant women with high risk for GDM
Settings: outpatient
Intervention: Exercise

Outcomes	Illustrative comparative risks* (95% CI)		Relative effect (95% CI)	No of Participants (studies)	Quality of the evidence (GRADE)	Comments
	Assumed risk	Corresponding risk				
	**Control**	**GDM**				
**GDM**	**Study population**		**OR 0.64** (0.51 to 0.8)	2742 (13 studies)	⊕⊕⊕⊖ **moderate**	
	**197 per 1000**	**136 per 1000** (111 to 164)				
	**Moderate**					
	**216 per 1000**	**150 per 1000** (123 to 181)				

*The basis for the **assumed risk** (e.g. the median control group risk across studies) is provided in footnotes. The **corresponding risk** (and its 95% confidence interval) is based on the assumed risk in the comparison group and the **relative effect** of the intervention (and its 95% CI).

CI: Confidence interval; **OR:** Odds ratio;

GRADE Working Group grades of evidence
High quality: Further research is very unlikely to change our confidence in the estimate of effect.
Moderate quality: Further research is likely to have an important impact on our confidence in the estimate of effect and may change the estimate.
Low quality: Further research is very likely to have an important impact on our confidence in the estimate of effect and is likely to change the estimate.
Very low quality: We are very uncertain about the estimate.

Table S14. GRADE evaluation of overall evidence for diet plus exercise intervention

Diet plus exercise for GDM prevention

Patient or population: pregnant women with high risk for GDM
Settings: outpatient
Intervention: Diet plus exercise

Outcomes	Illustrative comparative risks* (95% CI)		Relative effect (95% CI)	No of Participants (studies)	Quality of the evidence (GRADE)	Comments
	Assumed risk	Corresponding risk				
	Control	GDM				
GDM	**Study population**		OR 0.7 (0.55 to 0.9)	7673 (18 studies)	⊕⊕⊖⊖ low[1,2]	
	180 per 1000	133 per 1000 (108 to 165)				
	**Moderate**					
	255 per 1000	193 per 1000 (158 to 236)				

*The basis for the **assumed risk** (e.g. the median control group risk across studies) is provided in footnotes. The **corresponding risk** (and its 95% confidence interval) is based on the assumed risk in the comparison group and the **relative effect** of the intervention (and its 95% CI).

CI: Confidence interval; **OR:** Odds ratio;

GRADE Working Group grades of evidence
High quality: Further research is very unlikely to change our confidence in the estimate of effect.
Moderate quality: Further research is likely to have an important impact on our confidence in the estimate of effect and may change the estimate.
Low quality: Further research is very likely to have an important impact on our confidence in the estimate of effect and is likely to change the estimate.
Very low quality: We are very uncertain about the estimate.

[1] Significant level of heterogeneity
[2] Existence of publication bias
